# Silicon-Induced Tolerance against Arsenic Toxicity by Activating Physiological, Anatomical and Biochemical Regulation in *Phoenix dactylifera* (Date Palm)

**DOI:** 10.3390/plants11172263

**Published:** 2022-08-31

**Authors:** Taimoor Khan, Saqib Bilal, Sajjad Asaf, Safiya Salim Alamri, Muhammad Imran, Abdul Latif Khan, Ahmed Al-Rawahi, In-Jung Lee, Ahmed Al-Harrasi

**Affiliations:** 1 Natural & Medical Sciences Research Center, University of Nizwa, Nizwa 616, Oman; 2 Division of Plant Biosciences, School of Applied Biosciences, College of Agriculture & Life Science, Kyungpook National University, 80 Dahak-ro, Buk-gu, Daegu 41566, Korea; 3 Department of Engineering Technology, University of Houston, Sugar Land, TX 77479, USA

**Keywords:** date palm, antioxidants system, silicon, arsenic toxicity, organic acids, ABA, SA

## Abstract

Arsenic is a toxic metal abundantly present in agricultural, industrial, and pesticide effluents. To overcome arsenic toxicity and ensure safety for plant growth, silicon (Si) can play a significant role in its mitigation. Here, we aim to investigate the influence of silicon on date palm under arsenic toxicity by screening antioxidants accumulation, hormonal modulation, and the expression profile of abiotic stress-related genes. The results showed that arsenic exposure (As: 1.0 mM) significantly retarded growth attributes (shoot length, root length, fresh weight), reduced photosynthetic pigments, and raised reactive species levels. Contrarily, exogenous application of Si (Na_2_SiO_3_) to date palm roots strongly influenced stress mitigation by limiting the translocation of arsenic into roots and shoots as compared with the arsenic sole application. Furthermore, an enhanced accumulation of polyphenols (48%) and increased antioxidant activities (POD: 50%, PPO: 75%, GSH: 26.1%, CAT: 51%) resulted in a significant decrease in superoxide anion (O_2_^•−^: 58%) and lipid peroxidation (MDA: 1.7-fold), in silicon-treated plants, compared with control and arsenic-treated plants. The Si application also reduced the endogenous abscisic acid (ABA: 38%) under normal conditions, and salicylic acid (SA: 52%) and jasmonic acid levels (JA: 62%) under stress conditions as compared with control and arsenic. Interestingly, the genes; *zeaxanthin epoxidase* (*ZEP*) and *9-cis-epoxycarotenoid dioxygenase (NCED-1)* involved in ABA biosynthesis were upregulated by silicon under arsenic stress. Likewise, Si application also upregulated gene expression of plant plasma membrane ATPase (*PMMA-4*), aluminum-activated malate transporter (*ALMT*) responsible for maintaining cellular physiology, stomatal conductance, and short-chain dehydrogenases/reductases (*SDR*) involved in nutrients translocation. Hence, the study demonstrates the remarkable role of silicon in supporting growth and inducing arsenic tolerance by increasing antioxidant activities and endogenous hormones in date palm. The outcomes of our study can be employed in further studies to better understand arsenic tolerance and decode mechanism.

## 1. Introduction

Heavy metals (HMs) contamination is one of the several factors limiting sustainable crop productivity across agricultural ecosystems, caused by industrial effluents and domestic wastewater [1,2]. Currently, excessive HMs accumulation in food crops and toxicity in humans by entering food chains is a serious concern for researchers [1,2,3]. Excessive availability of toxic metals in soils significantly retards plant growth by inhibiting different metabolic and biochemical activities and leading to cell death [4,5]. Moreover, heavy metals contamination remarkably hampers the uptake and translocation of essential elements [6] and thus leading to morphological and physiological defects in plants by inducing necrosis in leaves, roots browning, enzymatic disruption, molecules degradation, and abnormalities in metabolic pathways that ultimately inhibit plant growth [3,4,5,7]. Besides, heavy metals contamination can also negatively affect the qualitative growth, yield, and safety of crops, further endangering food security [6]. 

Arsenic is categorized as an utmost lethal metalloid by the international agency for research on cancer as a primary carcinogen [8]. Previously, increased levels of arsenic generated naturally or by anthropogenic activities (mining, insecticides, fertilizers applications, and industrial wastes) have been reported in several countries throughout the globe, including India [9], Bangladesh [10] and China [11]. It exists in the environment in organic but more dominantly in inorganic forms as reduced trivalent arsenite (As III) or fully oxidized pentavalent arsenate (As V) [8]. While in soil, its bioavailability is based on comparative features of soil such as texture, pH, redox status, concentration of iron (Fe) and plant-available phosphorus, and its interactions with other elements [12,13,14]. However, increased mining of ores, employing arsenic-contaminated water for irrigation and ruminated usage of arsenic-planted pesticides (calcium arsenate, copper arsenate, and lead arsenate) ultimately increase the leftover As in soils [15,16]. Increased levels of As and its prolonged and prolonged exposure can severely affect plants and humans [4].

Plants can absorb As from water or soil via roots where excessive accumulation drastically affects cellular proliferation and roots elongation compared to above-ground parts of plants [6]. Moreover, it also hinders the photosynthetic system and raises functional damages in plants, making crops less productive because of high levels of As accumulation [17,18]. However, several approaches have been used for enhancing plant resistance and tolerance to withstand agro-ecosystem contamination of toxic materials including heavy metals (HMs). Application of plant growth-promoting elements for limiting HMs in soils or their translocation into plants is also a cost-efficient and eco-friendly approach [7,8,9,19]. In this regard, silicon (Si) has been reported as a growth-supporting element for plants and performs a key role in defending plants under detrimental conditions by stimulating their stress tolerance [20]. The growth-stimulating role and positive effects of Si have been previously verified in cotton [21], mustard [22], date palm [23], and wheat [24]. Mechanistically, Si can alleviate the toxic impacts of metallic contamination in plants by precipitation of metals, reducing their bioavailability, restricting their uptake and translocation via regulating HM-related transporter gene expression [25,26,27]. It also supports plant developmental growth by provoking enzymes and metabolites of the defense system, inducing structural adaptations in plants along with the chelating of metals [27]. Previous studies reported that silicon also promotes root exudates and limits metal uptake by chelation with phenolic-flavonoids or with organic acids [25]. It also showed influential interaction with phytohormones including abscisic acid (ABA), salicylic acid (SA), and jasmonic acid (JA), which are involved in promoting physiological and biochemical processes in plants responding to stress conditions [10]. For instance, several studies also demonstrated the reduction in As toxicity with phytohormones through an improved antioxidant system [11,12,13]. Moreover, it can improve cellular or tissue stability by accumulating at spines as amorphous silica (SiO_2_) and intercellular sites (phytoliths) to overcome oxidative stress [28]. Overall, its mechanism of action thoroughly relates to the accumulation of the reduced metal in plants, verified recently in the durum wheat [29], banana [30], cowpea [31], and rice [32], investigated under Cd, Pb, Al, and Cr metallic stressed conditions, respectively.

*Phoenix dactylifera* (date palm) is an endemic plant to gulf soils familiar with many applications as a food crop. It can be found globally in arid and semi-arid zones, particularly in Arab countries with significant cultivation and production levels. Oman stands at 8th in the list of countries with the highest global production of date palm with an average of >250,000 metric tons of annual production [5,33]. Among fruit crops in Oman, its cultivation covers more than 50% of total arable land with an above 80% growth rate. However, the environmental fluctuations, increased pesticides, and contaminated water induce severe stress on date palm [34,35]. Irrigating date palm fields with sewage water is the major reason for inducing heavy metal (HM) contamination and toxicity in plants, thereby leading to growth inhibition. [36]. Even though date palm has a certain level of stress tolerance, the overall growth, productivity, and quality of fruits are affected by many factors including heavy metals contamination. Studies have also shown the extensive capacity of date palm for HMs removal from wastewater, particularly for cadmium (Cd) and chromium (Cr) accumulation [37,38]. Previously, it has been explored for phytoremediation and adsorption of HMs; however, with detrimental consequences [39]. The excessive accumulation and exposure of date palms to HMs can harm their growth and fruit quality [40]. Mesnoua, et al. [41] reported the interaction of date palm with several heavy metals and found Cd highly toxic by prohibiting pollen grain germination and pollen tube elongation. For instance, aluminum (Al) has been reported to negatively influence embryogenesis in its callus cultures, affecting biochemical and molecular activities [42]. Moreover, date palm has been exploited for investigating its interaction with HMs such as Al, Cd, and Cr along with exogenously applied elements, where HMs were found with severe effects on its photosynthetic system, cellular defects, and biochemical alterations [23,43].

Therefore, it is crucial to prevent HMs toxicity in date palm in order to ensure its productivity and safety for humans feeding on it. So, the current study investigated the phytotoxicity and genotoxicity induced by arsenic trioxide in date palm by analyzing physiological and anatomical adaptations, and biochemical and molecular responses. Therefore, we aimed to assess the effect of silicon on the modification of plant root and shoot anatomy for the subsequent arsenic uptake, translocation, and their consequent effects on oxidative stress at biochemical and molecular levels as well as to illustrate stress-related hormonal interaction (ABA, SA, JA) and stress-responsive genes modulation by silicon supplementation under arsenic stress. Moreover, the study also aims to investigate organic acid regulation in plants by silicon intervention to ameliorate arsenic toxicity and improve essential nutrient regulation.

## 2. Material and Methods

### 2.1. Plant Growth and Treatment with Silicon and Arsenic

*Phoenix dactylifera* L. Khalas (date palm) seedlings (4 months old) were obtained from Agriculture Research Center in Bahla, Oman. The seedlings were replaced within pots (10 × 9 cm) having sphagnum peat moss (pH 4.5–5.5, bulk density 0.7–1.0 mgm^−3^, water content 38.5%, grain size 125–250 μm, electrical conductivity 2.0 dSm^−1^, organic matter 91.1% *w*/*w*, (nitrogen 800–2500 mg kg^−1^, phosphorus 150–850 mg kg^−1^, and NaCl (850 mg kg^−1^). Before applications of metal stress, the seedlings were placed in a greenhouse concealed by controlled growth conditions (temperature 30 ± 2 °C for 16/8 h (light/dark) photoperiod at relative humidity; 60–65%) for one week to acclimatize to the required growth conditions. All the seedlings were well watered on a daily basis. Later, the seedlings with uniform growth were selected for the experimental part by randomly arranging them into four different groups; group 1 CK plants (control: only dH_2_O), group 2 Si- plants (treated with silicon only), group 3 Si + AS plants (treated with silicon and arsenic), and group 4 (treated with arsenic only) and placed in a greenhouse under controlled growth conditions as mentioned above. The arsenic solution (1 mM, AsO_3_) was also prepared in distilled water and the pH was adjusted to 6.2–6.5. Silicon solution (1 mM), by dissolving Na_2_SiO_3_ in dH_2_O, was prepared and the pH was fixed to 6.2–6.8 by using NaOH (0.1 N) and HCL (0.1 N). The Si solution (100 mL) was poured into every pot of group 2 and 3 for 30 days. The control and group 4 were supplemented with dH_2_O only. Finally, after Si treatments, the plants of respective groups were exposed to As stress for 50 days. About 100 mL of each solution (Si: 1 mM), (As: 1 mM) and dH_2_O (100 mL) was applied daily to relevant pots with caution to reduce the chances of leaching. After recording data for several morphological parameters, the seedlings were directly harvested in liquid N_2_. The plants were immediately reserved in a deep freezer (−80 °C) for further investigations. 

### 2.2. Quantification of Photosynthetic Pigments

A briefly improved protocol by Sumanta, et al. [44] was employed to determine chlorophyll content. The fresh leaves of experimental date palm seedlings were cut and immediately ground with acetone (80%). The resultant mixture was centrifuged quickly for 5 min (at 12,000 rcf) to get clear supernatant. Thereafter, the supernatant was taken and the absorbance was recorded with a spectrophotometer at the respective wavelengths (663 nm, 645 nm, and 470 nm) for chlorophyll *a*, chlorophyll *b*, and carotenoids. 

### 2.3. Determination of Leaf Water Content 

To determine leaf relative water content in treated and non-treated plants, the protocol of Cao, et al. [45] was employed after slight alterations. Briefly, the fresh leaves of date palm seedlings were cut out and fresh mass (FM) was recorded. The leaves were then placed in distilled water (30 mL each) in Petri dishes and incubated for around 6 h. The turgid mass (TM) of leaves was recorded and water was removed. Finally, dry mass (DM) was noted after heat-treating the leaves at 80 °C in an incubator. To calculate relative water content (RWC), the following formula was employed.

RWC [%] = [(FM − DM)/(TM − DM)]/100.

### 2.4. Evaluation of Protein Content and Antioxidant Enzymes

The protocol mentioned by Bradford [46] was employed with minor improvements to determine protein contents. The leaves of date palm were powdered in a chilled mortar and pestle using potassium phosphate buffer (100 mM, pH 6.8) with ethylenediaminetetraacetic acid (EDTA: 0.2 M) and centrifuged for 25 min (at 12,000× *g*) to get the resultant crude mixture. The supernatant was measured by taking the absorbance value (595 nm). 

Furthermore, the anti-oxidative enzymatic assays: catalase (CAT), peroxidase (POD), polyphenol peroxidase (PPO), and total polyphenols (TPP), were determined by a protocol modified previously by our research group [23], with minor modifications. Briefly, liquid nitrogen was applied over leaves and effectively ground with a mortar and pestle. One hundred micrograms of the powdered sample was taken to mix with phosphate buffer (100 mM, pH 7.0) and mixed gently. The reaction mixture was centrifuged for 30 min at 10,000 rpm, 4 °C, and the upper layer was employed for further analysis.

For determination of peroxidase (POD) activity, 100 µL of samples extract was mixed with potassium phosphate buffer (0.1 M; pH 6.8), 50 µL H_2_O_2_ (50 mM) and 50 µL of pyrogallol (50 mM), followed by an incubation period of 5 min at 25 °C. The enzymatic reaction was stopped by adding H_2_SO_4_ (50% *v*/*v*), and the absorbance of the reaction mixture was measured at 420 nm. A similar reaction mixture for POD activity determination, excluding H_2_O_2_, was used by measuring the absorbance value at 420 nm. 

For instance, for the determination of catalase (CAT) activity, the protocol was employed as reported by Bilal, et al. [47]. Shortly, the sample extract was mixed with H_2_O_2_ (0.2 M) in 10 mM phosphate buffer (pH 7.0). After that, the activity was measured as a decrease in absorbance at 240 nm and expressed as units (one unit of CAT was defined as the ng of H_2_O_2_ released/mg protein/min).

### 2.5. Determination of MDA Levels

The protocol of Okaichi, et al. [48] was used to determine the formation of MDA or level of lipid peroxidation in date palm leaves, with minor changes. The leaves samples (100 mg powdered) were mixed in 10 mM phosphate buffer (pH 7.0) and centrifuged for 15 min at 12,000 rpm. The supernatant (0.2 mL) was mixed with 0.2 mL of sodium dodecyl sulfate (SDS; 8.1%), 1.5 mL of 20% acetic acid (pH 3.5), and 1.5 mL of 0.8% thiobarbituric aqueous acid (TBA) solution in a reaction tube. Then, the reaction mixture was warmed up to 90 °C for 1 h, followed by cooling down at 25 °C, and mixed with 5 mL of butanol: pyridine (15:1 *v*/*v*). Finally, after the appearance of pink color, the upper layer was taken and the absorbance was measured at 532 nm. Tetramethoxypropane was used as an external standard.

### 2.6. Determination of Superoxide Anion (O_2_^•−^)

The method of Gajewska and Skłodowska [49] was used to estimate the level of superoxide anion O^−2^ in date palm exposed to arsenic stress, with slight modifications. Briefly, 1 g of plant powder was mixed in 10 mM phosphate buffer (pH 7.0) containing nitrobluetetrazolium (NBT: 0.05% *w*/*v*) and sodium azide (NaN_3_: 10 mM) and incubated at 25 °C−30 °C for 1 h. Then, the reaction mixture was transferred into another tube and warmed at 85 °C for 15–20 min, then cooled to normal temperature and vacuum filtration. For instance, absorbance was measured at 580 nm with a spectrophotometer. The experiment was replicated thrice.

### 2.7. Elemental Analysis by Inductively-Coupled Plasma–Mass Spectrometry (ICP–MS)

The elemental analysis was performed using Bilal, et al. [50] developed protocol. Briefly, 1 g of leaves, roots, and soil samples each was taken to screen respectively and measure the concentration of elements: K, Ca, Mg, and Si. The samples were dissolved immediately in 4 mL of nitric acid (65%) and digested within ultra-microwave followed by dilution with dH_2_O to raise the final volume to 50 mL. The calibration curves were drawn with relevant standards, and the mixtures were then screened with ICP–MS (PerkinElmer Optima 7900 DV, PerkinElmer, Inc., Waltham, TX, USA).

### 2.8. Microscopic Analysis of Date Palm Shoot and Root Samples

An established procedure was followed for scanning electron microscopic analysis of samples, with slight modifications [51]. Fresh shoots and roots of date palm seedlings from normal and treated conditions were taken and stored in Formalin Acetic acid (3.7% *v*/*v* formaldehyde, 50% ethanol, 5% acetic acid) as a primary fixative. Then, under a stereoscope, the samples were properly dissected and washed twice with sodium cacodylate buffer (pH: 6.5, 0.1 M) for 20 min, followed by secondary fixation with osmium tetroxide (OsO_4_: 1%) for 1 h. Then, cross-sections were washed with dH_2_O, followed by a series of alcoholic treatments (ethanol: 30%, 50%, 70%, 90%, and 100%, three times each), to fully dehydrate the samples. A critical point dryer (SYSGLCP-8, Sanyu-Gijutsu, Akiruno, Japan) with a flow rate of 1.0 L/min at 40 °C was used to dry samples and remove ethanol with liquid CO_2_. The properly dried samples were then mounted on 10 × 10 aluminum stubs, gold-coated for 3 min, and screened with a scanning electron microscope. Subsequently, the SEM-EDS (JSM-6490LV, JEOL Ltd., Tokyo, Japan) was utilized to estimate the level of arsenic and silicon in root and shoot samples. The particulate areas of SEM images were analyzed using EDS to obtain atomic percentages of Si and As or any other elements that exist in samples.

### 2.9. Phytohormone Extraction and Quantification

For extraction of endogenous ABA, the methods of [50,52] were employed with little change. In brief, plant powder extract was subjected to a gas chromatography-mass spectrometry (6890 N network GC system and 5973 network mass selective detector; Agilent Technologies, Palo Alto, CA, USA) with ABA-[(±)-3,5,5,7,7,7-d6] supplemented as an internal standard. The spectra were recorded in selected ion mode at *m*/*z* 162 and 190 for Me–ABA and *m*/*z* 166 and 194 for Me–[^2^H^6^]–ABA. The ABA content was estimated by correlating the endogenous peaks and corresponding standards. Overall, three experimental replicas were used each, and the experiment was reproduced thrice. 

The optimized protocol reported previously was followed with few modifications for JA extraction and quantification [53]. Shortly, the same system was employed to screen samples with GC-MS, as described above. The fragment ion (*m*/*z* 83) was interpreted relatively with JA base peaks and [9,10–^2^H_2_]-9,10-dihydro-JA. Finally, the concentration of JA was estimated by correlating the endogenous JA peaks and respective standards.

Similarly, the extraction of salicylic acid (SA) was performed by utilizing powdered samples of date palm according to the protocol previously optimized by our research group [23,54] with slight changes. In brief, high-performance liquid chromatography (HPLC) was performed with an outfitted Shimadzu device having a fluorescence indicator (Shimadzu RF-10AxL), excitation at 305 nm and emission at 365 nm, and a C18 reverse-phase HPLC column (HP Hypersil ODS, particle size 5 µm, pore size 120 Å, Waters, Milford, MA, USA). The flow rate was maintained at 1.0 mL/min.

### 2.10. Organic Acids Extraction and Quantification

The methods reported by [52] were used to isolate and determine citric acid, malic acid, succinic acid, lactic acid, and acetic acid levels in plant samples. In brief, the plant samples were analyzed with high-performance liquid chromatography (HPLC) employing a Shimadzu device outfitted into a fluorescence detector (Shimadzu RF-10AxL) and a C18 reverse-phase HPLC column (HP Hypersil ODS, particle size 5 μm, pore size 10 Å; Waters). The respective wavelengths for excitation and emission were adjusted to 305 and 365 nm while the flow rate was set at 0.6 mL min^–1^. Three experimental replicas were employed, and the procedure was repeated thrice. 

### 2.11. RNA Extraction and cDNA Synthesis

The optimized protocol of Liu et al. was adopted with slight improvements to extract RNA and prepare cDNA [55]. Briefly, Tris-HCL (0.025 M, pH: 7.5) was prepared and fortified with NaCl (0.25 M), EDTA (20 mM), 1% *w*/*v* SDS and 4% *w*/*v* polyvinyl pyrrolidone. β-ME (0.1%) and ascorbic acid (0.5%) along with spermidine (0.1%) were added to the buffer solution just before sample addition. After that, fine powder (100 mg) was cautiously transferred to 2 mL RNase-free micro tubes having extraction buffer (750 µL) and immediately mixed with chloroform:isoamyl alcohol (CI; 24:1 *v*/*v*) in an equal volume. The reaction mixture was incubated (37 °C for 10 min) at 5 min intervals for short vertexing and then centrifuged (12,000× *g*, 4 °C for 10 min) to collect the supernatant (600 μL) in a new 2 mL tube. An equal volume of PCI (phenol:chloroform:isoamyl alcohol; 25:24:1 *v*/*v*) was added with the supernatant in all tubes, respectively. The solutions were mixed gently and centrifuged (12,000× *g*, 4 °C for 10 min). The upper layer (clean and clear) was then transferred to another (1.5 mL) microcentrifuge tube, and 1/10 volume of sodium acetate (3 M, pH 5.2) was added. For precipitation, absolute ethanol (two volumes) was also added. The tubes were kept for short inversions for proper mixing and incubated at 4 °C for 45 min. Later, after centrifugation (12,000× *g*, 4 °C for 10 min), the pellet was dissolved in DEPC-treated water (200 μL), mixed with lithium chloride (500 μL of 10 M), and kept at −20 °C for 60 min. Finally, the samples were centrifuged (12,000× *g*, 4 °C for 10 min) and the pellet was washed with 75% ethanol. The pellet was air-dried for 5 min and dissolved in TE buffer (50 μL). DNA was eliminated from RNA samples through DNase digestion by using Ambion DNase I (RNase free). Briefly, 1 µL DNase I buffer and 1 µL DNase I (2 U) were added to RNA samples and incubated for 10 min at 37 °C. After that, the RNA was quantified with NanoDrop and Qubit broad range kit (3.0), and quality was assured by observing bands on agarose gel electrophoresis with Gel-Doc.

For cDNA synthesis, the reaction mixture was prepared by using ten μL (>100 ng/μL) of extracted RNA and initially prepared Master Mix (comprised of RT buffer (2 μL), 25x dNTPs (0.8 μL), random primers (2 μL), reverse transcriptase (1 μL), and nuclease-free water (3.2 μL), in PCR tubes. The PCR reaction was run using a thermocycler with conditions adjusted as 25 °C for 10 min, 37 °C for 2 h, and 85 °C for 5 min. After the completion of the reaction, the cDNA was quantified with a Qubit DNA broad range kit and stored at −80 °C for molecular analysis. 

### 2.12. qRT–PCR Gene Expression Profiling

For evaluating expression patterns of stres-related genes (Table S1), the amplified cDNA was employed to perform qRT-PCR. Ubiquitin gene was applied as a reference/housekeeping gene. PCR reaction was performed with thermocycler using Power SYBR Green Master Mix and primers (forward and reverse 10 pM) for all genes of interest. The reaction was performed in triplicates for each sample to minimize contaminations and experimental errors. The PCR conditions were adjusted as follows: initially at 94 °C for 10 min, followed by 35 cycles at 94 °C (45 s), 65 °C (45 s), and 72 °C (1 min), while the extension step was performed at 72 °C (10 min). For amplification of genes, the threshold level was adjusted to 0.1. The reaction was repeated thrice with three replicas for each sample. 

### 2.13. Statistical Analysis

GraphPad Prism was used for all graphs designing and analysis of data (v7.02; San Diego, CA, USA). The data presented as the mean of all experimental replicas and ±SE. Duncan’s multiple range tests with a significant difference among treatments by ANOVA using SAS software (V9.1, Cary, NC, USA) to reveal significant to non-significant treatments by maintaining *p <* 0.05 and was represented by different lower-case alphabets 

## 3. Results

### 3.1. Effects of Silicon and Arsenic on Growth Attributes

**Figure 1:**
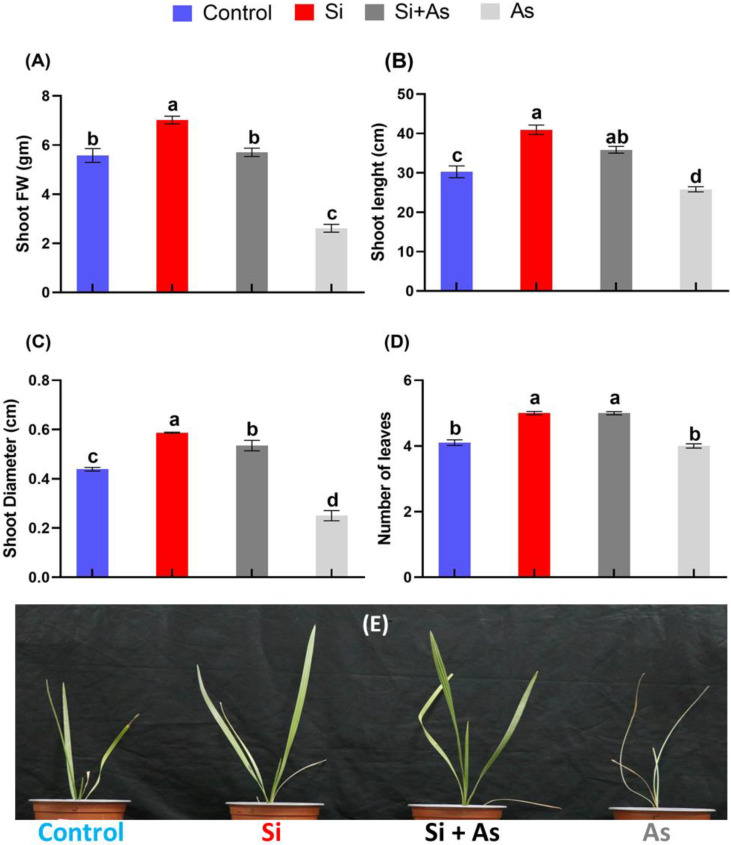
Morphological and physiological variations induced by heavy metal stress alone or in combination, in date palm. (**A**) Shoot fresh weight, **(B**) shoot length, (**C**) shoot diameter, and (**D**) number of leaves. (**E**) Date palm seedlings picture. Different letters indicate the values are significantly different *(p <* 0.05). Means were analyzed for finding the significant differences among treatments by using Duncan’s multiple range test (DMRT). Values represent means (of 7 replicates) ± standard error.

Current results revealed that silicon application improved plant growth under normal or As contamination. The As toxicity severely affected the development of roots and shoots of date palm after exposure of 50 days. However, shoot fresh weight of silicon-treated plants was found significantly increase (up to 26%) under control conditions (Si) and around 54% in stress conditions (Si + As) as compared with control (CK) and sole As-treated plants, respectively (Figure 1A). Likewise, the shoot length and diameter were also recorded maximum with respective increases (32% and 34%) by silicon alone or under arsenic contamination compared with CK plants (Figure 1B,C). Contrarily, the arsenic-treated plants expressed most minor biomass accumulation, and shoot fresh weight, length, and diameter significantly decreased (53%, 14.7%, and 43%) compared with control. Furthermore, the number of leaves per plant was also recorded higher in silicon-treated plants than in arsenic under both normal and stress conditions (Figure 1D). For instance, the leaves were morphologically found fresh, turgid, and greener in silicon-treated plants in both stress or average conditions, while sunken and drier under arsenic contamination (Figure 1E). 

**Figure 2:**
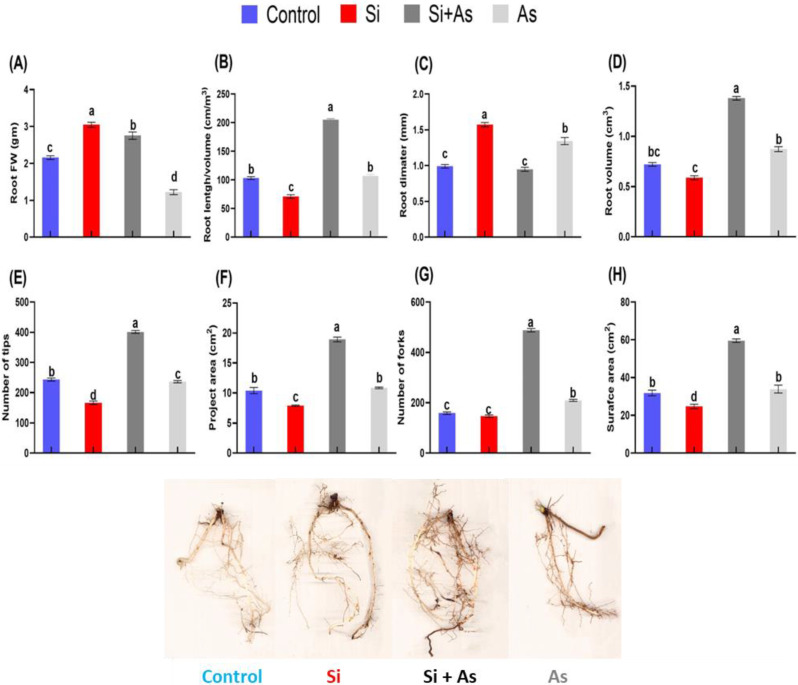
Variations in root architecture of date palm roots exposed to heavy metals stress. (**A**) Root fresh weight, (**B**) root length, (**C**) root diameter, (**D**) root volume, (**E**) number of tips, (**F**) project area, (**G**) number of forks, (**H**) surface area, analyzed with WinRHIZO image analysis system (Regent Instruments, Inc., Sainte-Foy, QC, Canada). Different symbols indicate the values are significantly different (*p <* 0.05). Means were analyzed for finding the significant differences among treatments by using Duncan’s multiple range test (DMRT). Values represent means (of 7 replicates) ± standard error.

Additionally, the growth attributes were further assessed in roots of date palm treated with Si and As. Results revealed that silicon significantly mitigated the toxicity generated by As. Fresh weight for roots was found higher (up to 41.3%) in plants treated with silicon (Si) than the in rest of the treatments (Si + As, As, and control) (Figure 2A). However, other attributes including root length volume and surface area along with project area and number of tips were recorded highest under silicon treatment in stressed conditions in comparison with sole As-treated plants (Figure 2B,D,E,H). The number of root tips and forks was recorded maximum under combined treatments of Si + As than control and silicon alone (Figure 2F,G). Furthermore, the morphological variations induced by all treatments are shown in (Figure 2).

Henceforth, the silicon was found to substantially reduce the toxicity of As by improving plant growth attributes. At the same time, sole arsenic application illustrated extended levels of toxic effects and reduced the overall growth of date palm. The results demonstrate the stress-mollifying aspect of silicon in date palm roots. 

### 3.2. Determination of Chlorophyll Contents and RWC

**Figure 3:**
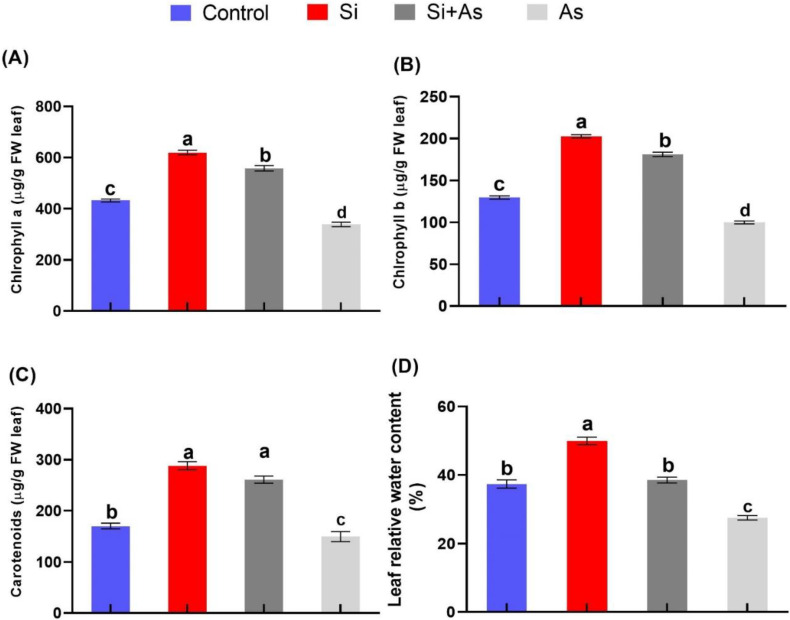
Influence of exogenously applied silicon (Si) on photosynthetic pigments in date palm seedlings under heavy metal stress conditions. (**A**) Chlorophyl a, (**B**) chlorophyl b, (**C**) carotenoids, and (**D**) relative water status. Different symbols show the significant difference among values (*p <* 0.05). Means for all treatment values were used to determine the significant difference by performing Duncan’s multiple range test (DMRT). Values represent means (of 7 replicates) ± standard error.

To assess biochemical investigations and develop an understanding of silicon fortified plant growth under arsenic contamination, photosynthetic pigments and relative water content in date palm were investigated. Surprisingly, the chlorophyll contents were found significantly (*p <* 0.01) enhanced in plants supplemented with silicon. For instance, the accumulation of chlorophyll a, chlorophyll b, and carotenoids were increased (up to 43%, 56%, and 67%, respectively) by silicon under normal conditions compared to control plants (Figure 3A–C). The sole arsenic-treated plants showed drastically reduced accumulation of photosynthetic pigments by approximately 54%, 62%, and 35% either alone or in combination with silicon as compared with combined Si + As-treated plants, sole Si-treated plants, and CK plants, respectively. Similarly, the RWC was also found to be highest (49.9 ± 2.5%) in silicon supplemented plants under normal conditions, with an increase of 33.5% as compared with control, and under stress conditions, Si-treated plants exhibited approximately 40% enhancement than sole As-treated plants (Figure 3D). Hence, silicon is proven to stimulate a protective role for photosynthetic pigments of date palms under arsenic stress conditions. 

### 3.3. Influence of Silicon on the Antioxidant System of Date Palm under Arsenic Stress

**Figure 4:**
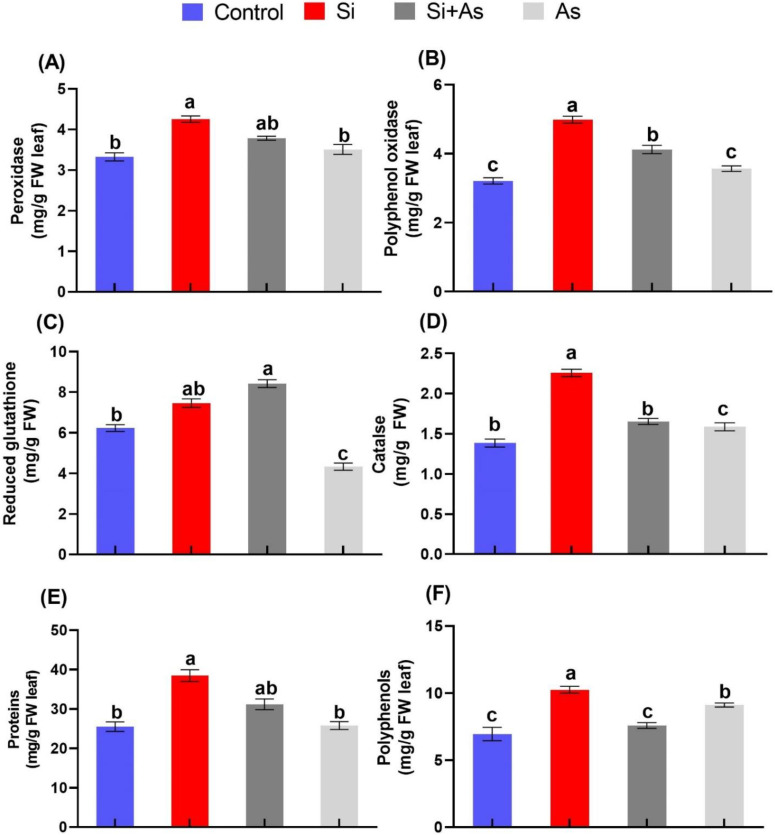
Stress alleviating effects of silicon (Si) exogenously applied, on antioxidants. (**A**) Peroxidase (POD), (**B**) polyphenol oxidase (PPO), (**C**) reduced glutathione (GSH), (**D**) catalase (CAT), (**E**) proteins contents, and (**F**) total polyphenol (TPP), in date palm under heavy metal stress conditions. Means for all respective treatments were used to determine significant difference (*p* > 0.05) via Duncan’s multiple range test (DMRT). Letters show significant difference and values represent mean (of 4 replicates) ± standard error.

The effects of Si and As on antioxidant enzyme activities in date palm are shown in Figure 4. Accordingly, results revealed that plants expressed significantly higher antioxidant activities under silicon feeding either alone or in combination with arsenic. Peroxidase activity under control conditions was insignificant between CK and Si-treated plants. However, a significant enhancement of 50% was found in silicon-supplemented plants under stress conditions than those of sole As-treated plants (Figure 4A). Likely, PPO activity was recorded with the highest induction by silicon with more than 75% relative increase than CK plants under control conditions (Figure 4B). While a substantial reduction in PPO activity by 17.1% was detected in sole As-treated plants in comparison with combined Si and As-treated plants. Furthermore, the GSH activity was found to be significantly enhanced in plants fortified with silicon under stress conditions. For instance, the GSH activity was shown by silicon-treated plants with a respective increase of 26.1% and 48.8% under stress conditions (Si + As) in comparison with CK and sole As-treated plants (Figure 4C). Moreover, the remarkable effects of silicon were further evaluated by measuring catalase enzyme activity in both treated and non-treated plants. A non-significant impact in CAT activity was detected between CK and Si-treated plants (Figure 4D). In contrast, Si application led to enhanced activity of CAT under As stress in comparison with sole As-treated plants by approximately 51%. Overall, the silicon neutralizes the toxicity induced by arsenic contamination via enhancing antioxidant enzyme functions. 

Moreover, plants treated with silicon expressed considerable levels of total polyphenols (TPP) and protein contents consistently with other antioxidant activities. The accumulation of total polyphenols (TPP) was found in silicon-treated plants with a significant increase of 48% under normal conditions; wherein, under combined treatment with arsenic, it was enhanced by around 10% (Figure 4F). Similarly, soluble proteins were detected maximum (38.4 ± 4.9 mg/g FW) in sole Si-treated plants, in comparison with other treatments. However, the levels of protein contents were significantly decreased by 44.8%, 51.3%, and 26.6% in sole As-treated plants when compared with combined Si and As treatment, only Si treatment, and CK treatment, respectively (Figure 4E).

### 3.4. Estimation of MDA and Superoxide Anion Levels

**Figure 5:**
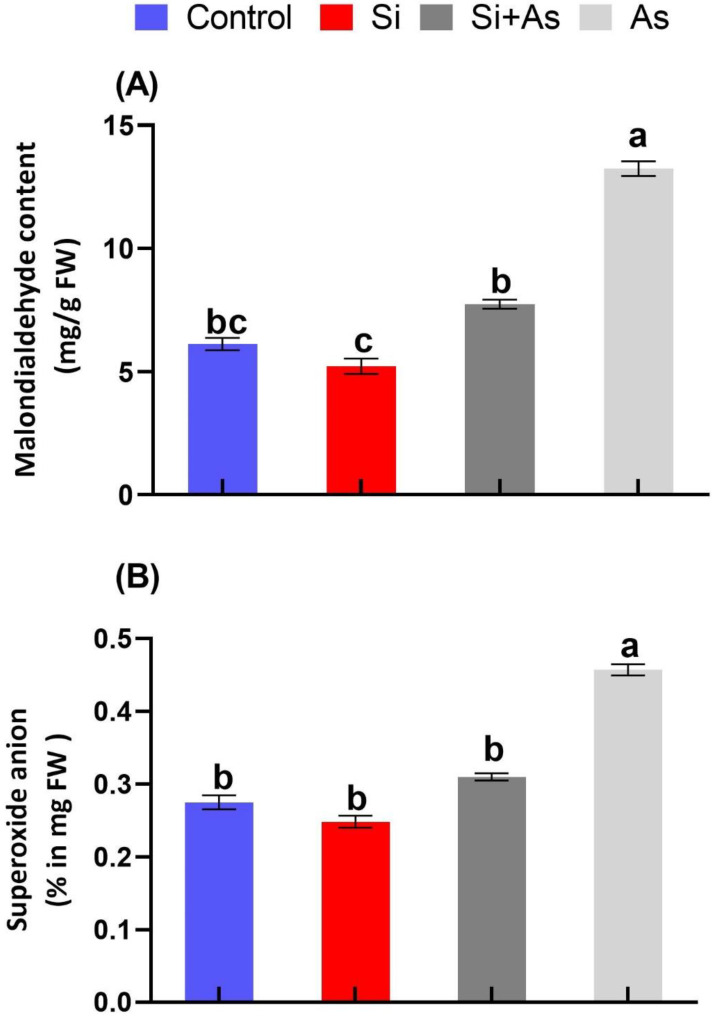
Stress-alleviating effects of silicon (Si) exogenously applied on (**A**) malondialdehyde (MDA) and (**B**) superoxide anion in date palm under heavy metal stress conditions. Means for all respective treatments were used to determine significant difference (*p* > 0.05) via Duncan’s multiple range test (DMRT). Symbols show significant difference and values represent mean (of 4 replicates) ± standard error.

The influence of arsenic stress and silicon treatments on membrane stability and lipid peroxidation was determined by investigating MDA accumulation in date palm (Figure 5). The stress induction by arsenic contamination gave rise to excessive reactive oxygen species (ROS) assemblies which caused significant MDA accumulation. This resulted in an increase of around 2.1-fold higher level of MDA in date palm exposed to arsenic stress in comparison to CK plants (Figure 5A). Contrarily, the toxic influence of arsenic was decreased by exhibiting around 1.7-fold lower level of MDA in comparison with sole As treatment, when plants were supplemented with silicon additionally. Similarly, the generation superoxide anion (O_2_^•−^) levels were recorded higher in stressed conditions than in normal conditions, considering stress affected membrane integrity. However, the O_2_^•−^ level was reduced by approximately 58% in plants treated with silicon in comparison with sole As under stress conditions (Figure 5B). These findings suggest the stress-alleviating role of silicon as evident by significant control of MDA and superoxide anion amounts in date palm.

### 3.5. Influence of Silicon on Phytohormones

**Figure 6:**
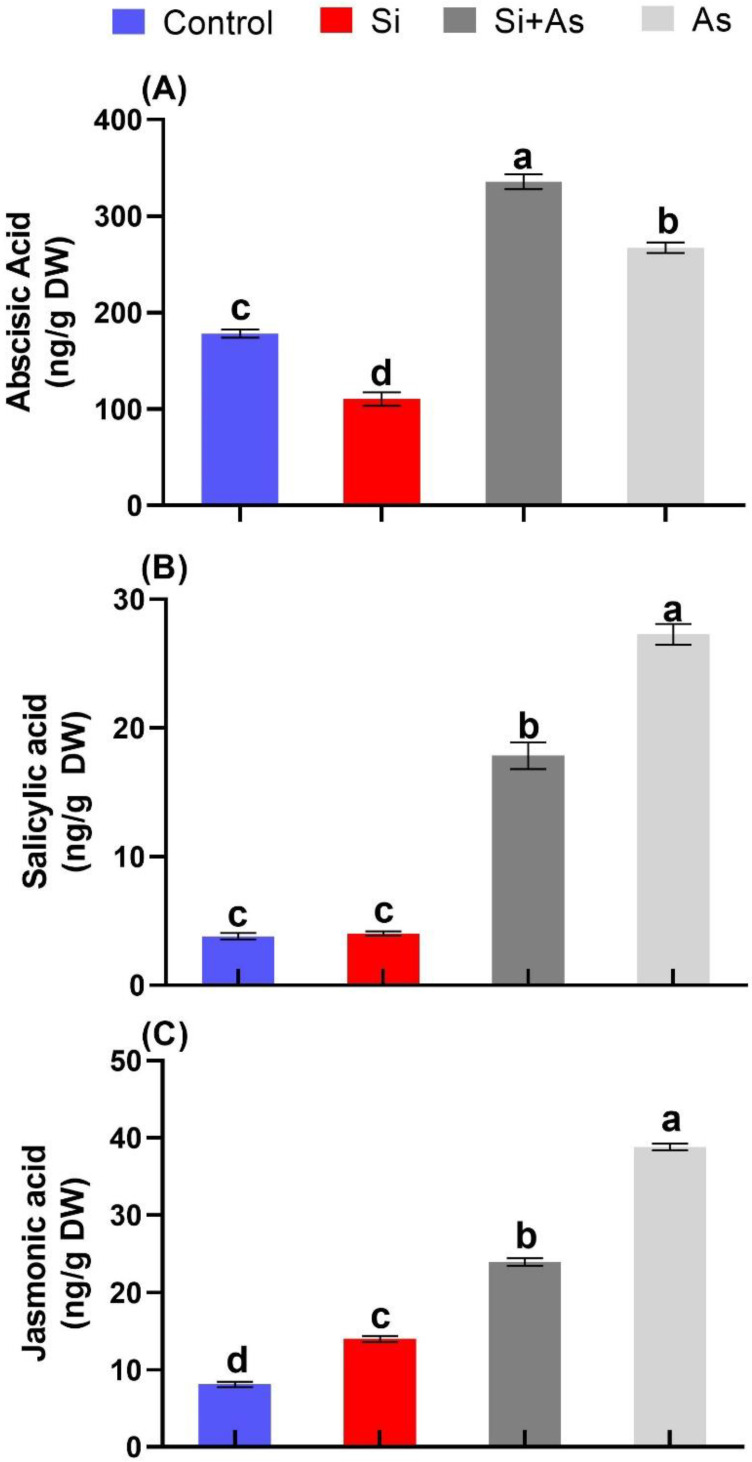
Effects of arsenic stress and silicon treatments on regulation of endogenous hormone, i.e., (**A**) abscisic acid, (**B**) salicylic acid, (**C**) jasmonic acid in date palm seedlings, treated with silicon and arsenic. Data represented as mean (4 replicas) ± shows standard error, symbols denote significant differences among values as determined by Duncan’s multiple range test (DMRT).

Plants produce stress-related hormones to cope with the toxicity generated by stress. The results showed that silicon also triggered this endogenous phytohormonal system’s expression and was significantly enhanced under stress conditions. Under normal conditions, plants treated with silicon expressed reduced ABA levels (38%) compared to CK plants (Figure 6A). However, the ABA level was significantly increased in combined Si and As-treated plants by 20%, 67%, and 46% compared to sole As-treated, sole Si-treated, and CK plants, respectively. The arsenic-supplemented plants also depicted significant amounts of ABA produced compared to control. The endogenous salicylic acid concentration was increased in stress conditions compared to control conditions (Figure 6B). However, significantly reduced accumulation of SA was recorded in plants under combined treatment of Si and As by 52% than those of sole As-treated plants. (Figure 6 B). The SA concentrations were not significantly variable in silicon sole treatments and control plants.

Similarly, the expression pattern for endogenous jasmonic acid was found to increase from normal to stressed conditions. The sole application of As caused a significant increase in JA content compared with CK plants (non-treated) (Figure 6C). The silicon application remarkably reduced the accumulation of JA under stress conditions by 62% compared with sole As-treated plants. In contrast, Si application significantly induced JA accumulation under control conditions compared to CK plants.

### 3.6. Modulation of Organic Acids Levels by Si

**Figure 7:**
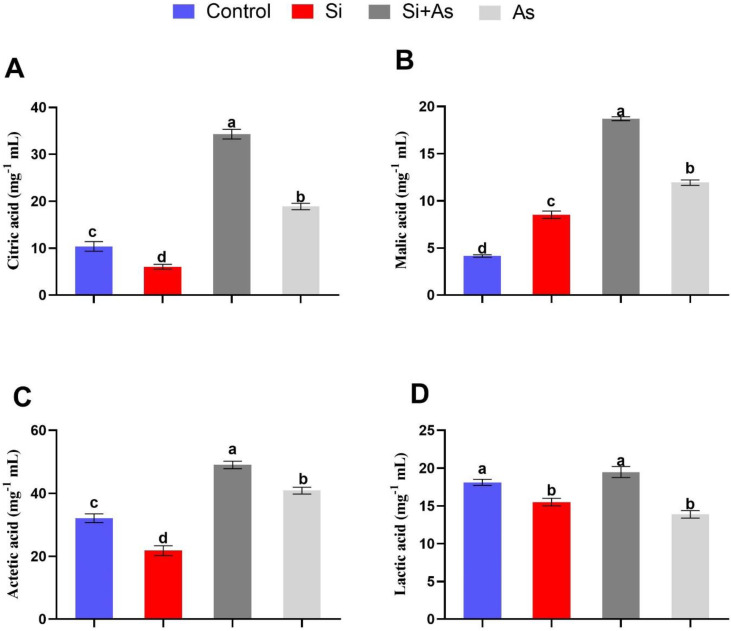
Effects of Si on organic acids regulation. (**A**) Citric acid, (**B**) malic acid, (**C**) acetic acid, and (**D**) lactic acid levels in date palm roots and soil, i.e., treated and non-treated with silicon and arsenic (Si, XSi, Si + As, and As). Data represented as mean (6 replicas) ± shows standard error, symbols denote significant differences among values as determined by Duncan’s multiple range test (DMRT).

Exogenous application of Si exhibited a varied effect on the regulation of organic acids (citric acid, malic acid, acetic acid, and lactic acid) in root under control conditions compared with CK plants by illustrating significantly reduced levels of analyzed organic acids except for malic acid (Figure 7). However, induction of As toxicity substantially affected organic acids regulation in sole As-treated plants compared with combined Si and As-treated plants by exhibiting significantly reduced levels of organic acids. Si-treated plants under As stress demonstrated approximately 1.82-, 1.57-, 1.2-, and 1.4-fold higher levels of citric acid, malic acid, acetic acid, and lactic acid than sole As-treated plants, respectively. 

### 3.7. Influence of Silicon on Plant Anatomy in Stress

**Figure 8:**
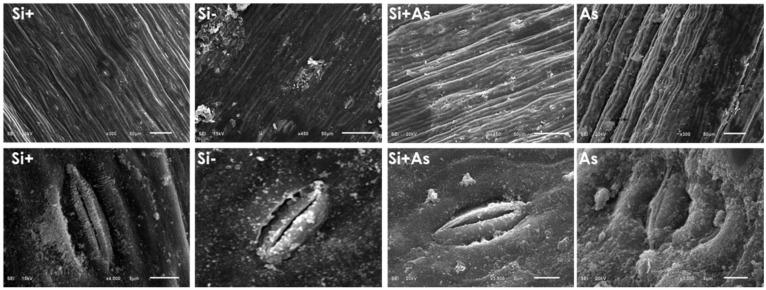
Physiological influence of silicon and arsenic on stomata apertures of date palm leaves, screened with scanning electron microscope. Silicon treated (Si+), silicon non-treated (Si−), silicon and arsenic (Si + As), and arsenic treated (As). Three biological replicates were screened at magnification 3500×, scale bar = 5 µm.

**Figure 9:**
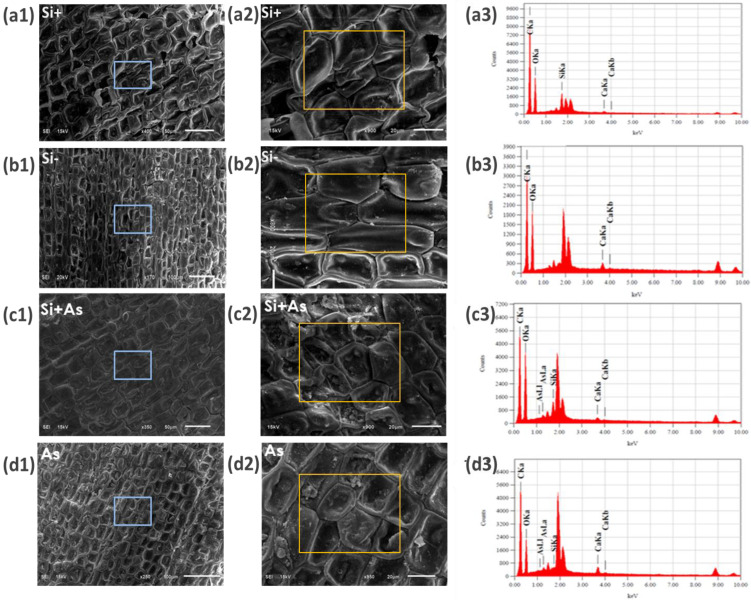
Scanning electron microscopy (SEM) images of date palm roots under different treatments of non-silicon (Si-), silicon (Si+), combined (Si + As), and arsenic (As). Images (**a1**,**a2**,**b1**,**b2**,**c1**,**c2**,**d1**,**d2**) represent morphological variations and element granules (circled blue and yellow), respectively, for Si+, Si-, Si + As, and As. The encircled areas in yellow were selected for EDS-based elemental analysis (**a3**) showing EDX-elemental peaks for Si-treated samples, (**b3**) (Si-), (**c3**) (Si + As), and (**d3**) represent data for As-treated plants, respectively. Three biological replicates were screened for each sample.

**Figure 10:**
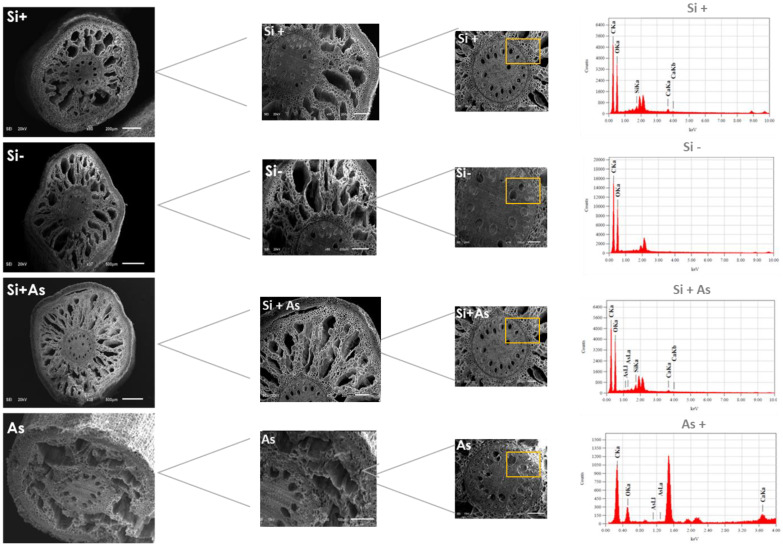
Scanning electron microscopy (SEM) images of date palm roots under different treatments of non-silicon (Si−), silicon (Si+), combine (Si + As), and arsenic (As). Images represent Si and As effects on roots anatomy from low to higher magnifications, respectively, for Si+, Si-, Si + As, and As. The encircled areas in yellow were selected for EDS-based elemental analysis showing EDX-elemental peaks for Si+, Si-, Si + As, and As-treated plants, respectively. Three biological replicates were screen for each sample.

To assess further the physiological effects of Si and As, date palm shoot and root samples were screened under a scanning electron microscope (SEM). In shoot samples, the influence of silicon and arsenic was variable on stomatal morphology. Whereas, enhanced stomatal density was found in Si-treated plants than in those of As and control (Figure 8). Likewise, the size of stomatal apertures was increased under silicon (Si+) supplementation and anatomical variations in leaves of treated and non-treated plants are mentioned in Supplementary Figures S1 and S2. The stomatal apertures were also observed to be smaller with a reduced opening under arsenic stress. For root samples, silicon applications enhanced cellular morphology, resulting in more compact epidermal layers under normal or stress conditions (Figure 9 and Figure 10). The SEM screening also showed that silicon-treated plants maintained endodermis, cortex, and pericycle along vascular bundles more in stress conditions than normal and control. Contrarily, arsenic-induced stress affected overall root morphology and was found injurious. 

The silicon and arsenic levels were further determined with SEM-EDS screening as shown in Figure 9 and Figure 10. Comparatively, the silicon was found higher by mass (7.63% and 2.36%, respectively) in leaves and roots treated with silicon only. Where plants treated with both silicon and arsenic showed respective weights of Si (7.6% and 3.17%) and arsenic (0.03% and 1.02%) in leaves and roots samples screened with SEM-EDS. In correspondence, no amount was detected for silicon or arsenic in control plants (-Si). Moreover, the arsenic fed plants accumulated 0.75% of arsenic in leaves and 3.09% in root samples. 

### 3.8. Determination of Endogenous Si and Arsenic Levels 

**Figure 11:**
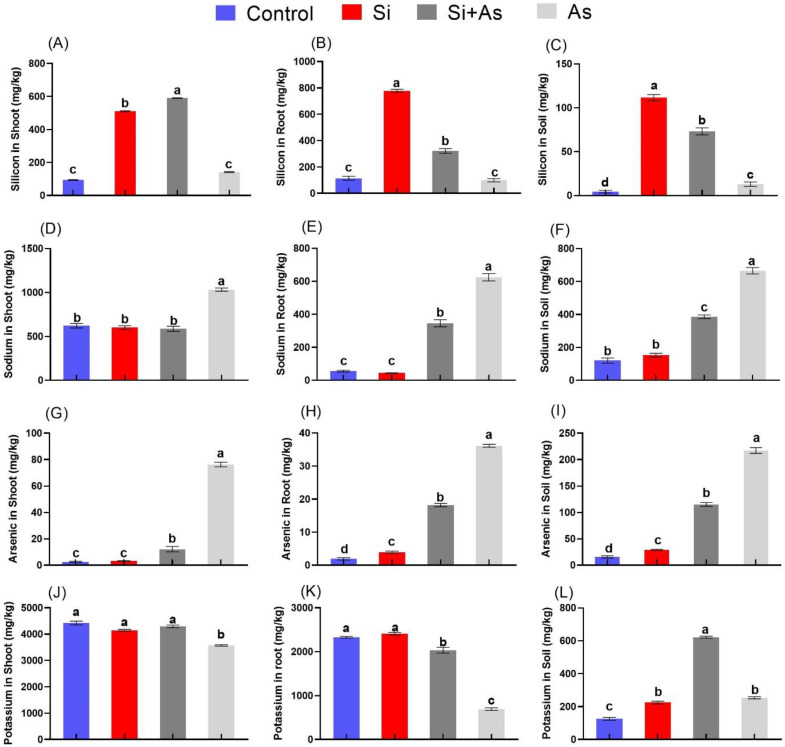
Elemental analysis of silicon (Si), arsenic (As) exogenously applied, and essential nutrients levels determinations via ICP in date palm. Means for all respective treatments were used to determine significant difference (*p >* 0.05) via by Duncan’s multiple range test (DMRT). Symbols show significant difference and values represent mean (of 4 replicates) ± standard error.

Date palm treated with Si and exposed to arsenic toxicity were screened with ICP-MS to determine endogenous levels of Si, As, Na, and K in shoot, root, and soil (Figure 11). Results showed that the highest accumulation for Si occurred in shoots under control conditions (511.4 mg kg^−^^1^) with an increase of >400%, compared with in the shoots of CK plants. Whereas significant endogenous Si accumulation (591 mg kg^−^^1^) was detected in the shoots of combined Si and As-treated plants with an increase of >300%, compared with sole As-treated plants. Subsequently, a significant level of Si in roots and soil was detected in sole Si-treated plants, followed by combined Si and As-treated plants (Figure 11A–C). Moreover, respective concentrations of arsenic in shoot (76.2 mg kg^−1^), root (36.1 mg kg^−1^), and soil (217.2 mg kg^−1^) were recorded highest in arsenic-fed plants only, followed by a marked reduction in plants treated with combined Si and As (Figure 11G–I). Likewise, Na and K concentrations were also measured, and a significant variation was recorded (Figure 11D–F,J–L)). Among treatments, Na concentration of shoot was found maximum in sole arsenic-treated plants, followed by other treatments with insignificant differences. The silicon application significantly reduced Na accumulation in roots, shoots, and soil compared to sole As-treated roots, shoots, and soil. Furthermore, the level of K was markedly enhanced in almost all treatments in shoot. Whereas in roots, the sole Si-treated plants demonstrated a reduced level by 70% compared to other treatments. Moreover, the accumulation of K in soil was significantly higher in combined Si treatment compared to other treatments.

### 3.9. Expression of Abiotic Stress-Associated Genes

**Figure 12:**
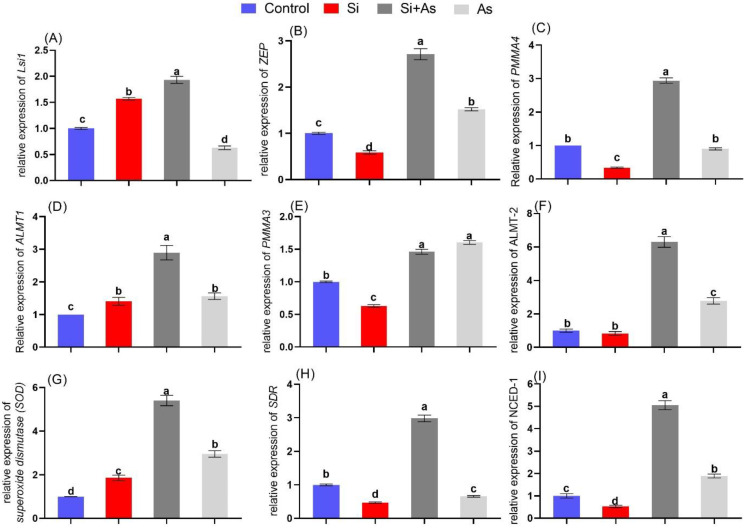
Influence of silicon on expression pattern of abiotic stress-related genes in date palm seedlings under metal stress. Data represented as mean ± standard error. The letters denote significant difference among treatments evaluated by Duncan’s multiple range test (DMRT).

The relative expression of genes linked with abiotic stress signaling and transportation of elements was investigated to investigate the underlying molecular mechanism of arsenic toxicity and silicon stress alleviation in date palm. The expression of the silicon transporter gene (*Lsi-1*) was relatively enhanced in plants treated with silicon under normal conditions. Under stress conditions, Si-treated plants exhibited approximately 3.1-fold higher transcript accumulation of Lsi-1 compared to sole As-treated plants (Figure 12A). Similarly, the *zeaxanthin epoxidase* (*ZEP*) involved in ABA biosynthesis was significantly downregulated in plants treated with silicon alone, and markedly upregulated in combined Si and As-treated plants. (Figure 12B). Similarly, the transcript accumulation of *PMMA-3* under stress conditions was insignificant. At the same time, *PMMA-4* of combined Si-treated plants revealed a significant upregulation by 3.4-fold under stress conditions compared with those of sole As-treated plants. (Figure 12C,E). Moreover, the arsenic stress induced a significant reduction in *ALMT-1* and *ALMT-2* expression in comparison with combined Si and As-treated plants (Figure 12D,F). Likewise, the antioxidants-related gene superoxide dismutase (*SOD)* higher expression and nutrients up-taking related gene short-chain dehydrogenase (*SDR)* displayed reduced expression in sole Si-treated plants under control conditions in comparison with CK plants. In the case of stress conditions, combined Si and As-treated plants illustrated around 1.8-fold and 4.5-fold higher transcript accumulation of *SOD* and *SDR* compared with sole As-treated plants (Figure 12G,H). The relative expression of *NCED*, a key protein encoder involved in ABA biosynthesis, was also significantly downregulated in sole Si-treated plants and markedly enhanced in combined Si and As-treated plants by 2.67-fold than those of sole As-treated plants under As stress (Figure 12I).

## 4. Discussion

The existence of heavy metals contamination in agricultural lands negatively affects plant productivity by hampering metabolic and biochemical processes and thereby inhibiting plant growth and development. The situation becomes further alarming, particularly during initial growth phases that lead to adverse developmental consequences [56]. Arsenic, a primary carcinogenic metalloid, also induces phytotoxicity and crop loss, despite being potent for human health [57]. To reduce arsenic stress, it is vital to investigate and develop strategies for limiting its uptake, accumulation, and translocation, particularly in food crops. Silicon was exogenously employed in this study to evaluate its interaction with date palm in reducing arsenic-induced toxicity, as previously reported for mitigating stress in several studies [58]. 

We found As with severe detrimental effects on date palm shoot and root growth. Arsenic affected cellular proliferation in shoot and root, retarded phototrophic, geotropic expansion, and induced necrosis. However, exogenous silicon applications promoted growth in normal or stressed conditions by supplementing mechanical strength. The morphological and growth parameters such as shoots elongation, leave width, number of leaves, roots length, and architecture were improved under silicon applications. The mitigating stress effects of silicon in date palm were more prominent in Si + As treatments as compared with As alone and control. Under As, the leaves were found dry and sunken, whereas silicon effectively maintained the turgidity and morphology of the plant under both normal and stress conditions. The possible reason behind silicon effects could be its potential mechanical strength under stressed conditions, which leads to optimum growth [26,59]. Similar effects of silicon in date palm for mitigating heavy metals stress were reported previously [23].

Similarly, the root biomass and phenotypic features were significantly improved with silicon feeding, while arsenic-induced severe stress on roots, resulting in brownish dry roots. The growth restoration and improving biomass of leaves and roots could be ascribed to the enhanced physiological features of silicon treatment in abiotic stress. Silicon also can promote tissue growth and boost plant systems mechanically under stress conditions [60]. Si is also reported to promote cellular or tissue stability against oxidative damage by strengthening the cell walls of leaves, roots, and stems [28]. 

The photosynthetic system was also investigated by analyzing chlorophyll contents (Chl a, Chl b) and carotenoids. The effects were found in correlation with morphological effects, and the contents were recorded to be reduced tremendously in plants exposed to arsenic stress solely. Such decline in photosynthetic pigments could be linked with disruption of membrane lipids by ROS generation, as toxicity influences δ-aminolevulinic acid dehydratase that negatively affects the biosynthesis of chlorophyll [61,62]. Moreover, the destruction of chlorophyll contents by arsenic stress is reported to lead to less CO_2_ assimilation and result in retarded growth under arsenic [63]. Contrarily, silicon-treated plants expressed increased photosynthetic components that aided in stress alleviation and improved carotenoid pigments under arsenic contamination. Silicon application is reported to perform the function of arsenic stress reduction by promoting green pigments initially and, at the same time, inhibiting the accumulation of arsenic in plant parts, which coincides with our current findings [4]. Moreover, it has been validated that silicon limits As uptake and inhibits its accumulation as both share the same transportation system [64,65]. Furthermore, the relative water content was also recorded enhanced in silicon supplemented date palm plants, as previously reported that Si also strengthens cuticle layers below the epidermis and limits water loss by controlled transpiration in stress environments [66]. Our results validated the same findings of silicon improving water levels and green pigments in different plant species under stress conditions [67,68,69].

Usually, the root is the first organ through which As enters, crossing the epidermis it passes through apoplastic/symplastic pathways to access the xylem and phloem and reach across different parts of the plant [14]. However, several plasma membrane associate components, selective transports, and pathways play significant roles in its entrance/uptake, translocation, and deposition [15]. As toxicity is also known for disrupting the nutrient homeostasis in plants by interfering with nutrient uptake and generating excessive ROS. Our results revealed that Si application effectively inhibited uptake and accumulation of As under normal or stress conditions. This could be accredited to the uptake and deposition of Si in roots and shoots, which subsequently limited As absorption and uptake. Such enhanced accumulation of Si is crucial for inducing tolerance to stress conditions by several mechanisms, including forming complexes to stabilize and control the exchange of destructive ions such as Na and metallic precipitations [28,70]. Mechanistically, As enters through root nodulin intrinsic proteins (NIP); together known as aquaporin channels. In rice roots, the expression of OsNIP2;1 (Lsi1) modulates the influx of silicic acid (Si(OH)_4_) and As(III), while OsNIP2;2 (Lsi2) serves as an Si(OH)_4_ efflux transporter across the cell membrane [16]. However, the process of Si(OH)_4_ efflux via *Lsi2* is restricted to the proximal side of the cell which inhibits the entrance of As [6]. In parallel, the increased deposition of Si in roots and its translocation to the ariel parts was found in Si-treated plants, which is known for inhibiting the passage of lethal cell-degrading ions, such as As, through forming complexes and the precipitation of metal ions as a co-factor. Our data also showed that silicon improved plants’ capacity for potassium uptake and translocation from roots to shoots, where maximum absorption occurred in shoots under normal and stressed conditions. This demonstrates the silicon’s ability to ensure nutrient homeostasis and efficient modulation of metabolic activities in stress conditions. However, further mechanistic studies are required to explore Si-based nutrient transportation, blocking metals and root activities, and the distinct influence of any nutrient among all available elements in soil. 

Subsequently, the plant defense system also showed a strong protective influence on silicon and arsenic toxicity. Arsenic uptake and accumulation severely induced oxidative stress and generated high levels of ROS and free radicals. These species cause damage to essential biomolecules such as lipids and disrupt metabolic function and cellular morphology. The level of membrane injuries is indicated by the amount of MDA detected as an end product of lipid peroxidation [71,72]. However, silicon is also reported to be involved with MDA, H_2_O_2_, and O_2_^•−^- metabolism, which are considered markers to evaluate ROS-induced membrane damages [73]. The exact mechanism of silicon interaction with these stress markers is not fully known. At the same time, several studies proposed the scavenging of ROS by silicon is achieved via provoked activation of the antioxidant defense system in plants under several abiotic stresses [74]. For instance, it is believed that Si also mediates plant defense by increasing phenolics, phytoalexins, chitinases, and other plant resistance genes [75]. In this study, arsenic exposure induced damage to the cell membrane resulting in increased MDA levels in arsenic-treated plants. Applications of silicon either alone or in combination with As reduced MDA levels by confining As translocation and neutralizing cell membrane damage through ROS scavenging under stress [61]. Plants also have developed several defense mechanisms, i.e., enzymatic (SOD, CAT, POD, PPO) and non-enzymatic (GSH), to cope with heavy metals-induced ROS by scavenging or reducing them. However, excessively exposed and high levels of stress induce deleterious effects on the defense system of plants by increased oxidative stress and ROS generation [76,77]. Herein, Si supported date palm growth by boosting antioxidant activities in stress, which was also evident from the upregulation of *SOD* expression. The elevated levels of soluble proteins and antioxidant enzymes could be linked with more ROS scavenging, thereby reducing MDA and O_2_^•−^. The findings demonstrate the protective role of silicon against ROS generated by As stress by provoking a string antioxidant system in date palm. Our data agree with the previously investigated role of silicon alone or in combination in different plants [78,79]. Overall, the Si mechanism of action thoroughly relates to the accumulation of the reduced metal in plants, verified recently in durum wheat [29], banana [30], cowpea [31], and rice [32], investigated under Cd, Pb, Al, and Cr metallic-induced stress conditions, respectively.

Plants activate defense-related endogenous hormonal systems to overcome the adverse effects of abiotic stresses. Current results showed that arsenic stress increased endogenous ABA levels in date palms. Plants treated with silicon under As stress appeared with significantly boosted levels of ABA content compared with control or sole As-treated plants. The recorded ABA levels were correlated with expression levels of *ZEP* and *NCED* genes, coding proteins involved in ABA biosynthesis [70,80,81,82]. Their expression was significantly enhanced with silicon application as compared with As. The current findings prove that application of Si under As-induced stress upregulated *ZEP* and *NCED* expression drastically to respond immediately to stress conditions. Thus, ABA biosynthesis is activated [83]. This could have led silicon-treated plants to maintain properly stomatal conductance and photosynthesis and improve the antioxidant system in date palm. The findings further affirm the role of silicon in managing stress conditions and maintaining plant developmental processes.

Furthermore, SA, an essential signaling molecule involved in maintaining plant growth under stress conditions, was also influenced by exogenous Si application in the current study. Herein, sole As application induced more SA accumulation than Si-treated plants. Such reduced biosynthesis of ABA under As stress in Si-treated plants could be ascribed to the upregulation of ABA biosynthesis, as an antagonistic interaction between SA and ABA accumulation has been reported [84]. Such reduced accumulation of SA could result from ameliorating heavy metal stress by silicon. Heavy metals-induced stresses are described for activating phenylalanine ammonia-lyase activities, which subsequently aid in the catalysis of chorismate-derived L-phenylalanine [85,86]. Hence the lower generation of SA in date palm under stress conditions indicates the decreased activity of chorismate-derived L-phenylalanine due to mitigation of As-induced stress. Consequently, a reduced level of SA resulted.

Furthermore, a similar trend was observed for JA accumulation among all treatments. As stress induced a high level of JA while silicon reduced its expression levels. Such Lower accumulation of JA could be attributed to stress ameliorating effects of Si by alleviating Si stress; thereby, less accumulation of JA was detected. The lower accumulation of JA could also be due to plant growth and biomass promotion by Si application and thereby leading to a higher accumulation of gibberellic acids, which can degrade DELLA proteins, and thus, jasmonate ZIM-domain (JAZs) proteins are attached with MYC2 for inhibiting its activity from reducing JA signaling [87].

To further understand Si and As interactions, the expression of several abiotic stress and transporter genes was evaluated with quantitative RT-PCR. The current results demonstrated that silicon and arsenic prompt distinct motifs of gene expression. The gene transcript responsible for the transportation of silicon (*Lsi-1*) was positively modulated in plants treated with silicon, regardless of control and stress conditions. The finding suggests that exogenous application of Si assisted date palm in accumulating Si via roots and permitted its translocation to shoots. Such significant accumulation in roots and translocation to shoots delivers additional strength to plants for encountering toxic heavy metals ([88]), as demonstrated in our micrograph after As stress. A significant deposition of Si was in endodermis and exodermis regions of date palm after As stress, resulting in less damage in the roots and shoots induced by As toxicity. Therefore, reduced As accumulation in shoot and roots was detected due to significant accumulation by activation of *Lsi* expression. 

Plant plasma membrane ATPases, as key enzymes mediating cellular physiology in plants under stress, are encoded by the PPMAase gene family [89]. They regulate pH, nutrient uptake, stomatal openings, and phloem loading [90,91]. Herein, the expression of *PMMA-4* was upregulated in plants treated with silicon under normal conditions. Wherein, plants under stress conditions expressed declined levels of *PPMA-4*. Heavy metals have a significant role in plant growth. However, several metals such as Cu, Zn, etc., become toxic in increased amounts. The plasma membrane is the first barrier HMs can damage, so PM-H+ ATPases generate a proton gradient needed to ensure ion homeostasis under stress [92]. However, the activities of *PMMA* are also reported to be modulated by several factors such as hormonal interactions and stress-related hormonal regulation [93]. Previously, Hu et al. (2020) described endogenous ABA regulation as a major factor in the detoxification of heavy metals, inhibiting their uptake and translocation and enhancing the conjugation with chelators. At the same time, ABA is also reported to trigger the transcript accumulation of plasma membrane *HMATPases* (*HMA3* and *HMA4*) for promoting heavy metals resistance, inhibiting uptake and translocation from roots to aerial parts in *Sedum alfredii* [94]. Therefore, the enhanced regulation of ABA in date palm by Si application could be interlinked with upregulation of *PMMA4* expression to limit As uptake and translocation to cope with As-induced toxicity. 

Furthermore, significant expression of *ALMT-1*, *ALMT-2* under As stress by Si application led to higher production of malic acids and citric acids in roots, which subsequently exudate to rhizosphere for inhibiting the entrance of metals into plant cells via the formation of stable complexes. Moreover, the detoxification of arsenic and inducing tolerance require removing metal ions or limiting its compartmentalization, which involves vacuoles or proteins with higher metal affinity [95,96]. Plants also utilize several enzymatic or non-enzymatic antioxidant series to accumulate ROS [97]. We recorded variable expression levels of SOD, however, enhanced in plants treated with silicon under arsenic stress than in sole silicon or arsenic application.

Short-chain dehydrogenases/reductases (SDRs) are members of the NAD-P-H-dependent oxidoreductase protein superfamily [98] and are well known for their remarkable role in primary or secondary metabolic activities, including biosynthesis of phenolics and alkaloids [99], fatty acids [100], steroids [101], and chlorophyll biosynthesis [102]. Subsequently, the secondary metabolites are generated and accumulated in the form of phytochemical defense under stress conditions. Herein, the higher expression of *SDR* by Si application under As stress indicates the modulation of defense-related metabolites regulation, including phenolic accumulation for alleviating As-induced toxicity. Hence, a positive correlation can be established between its expressions, generated increased phenolics, and expressed high antioxidant response under arsenic stress and silicon. 

Moreover, *SDR* genes are reportedly involved in defense-related hormonal modulation such as ABA and SA. Previously aAtSDR-1 as ABA deficient 2, catalyzed a key step in ABA biosynthesis by converting xanthoxin to abscisic aldehyde [103]. Likely, *AtSDR3* confers resistance to *Pseudomonas syringae pv.* tomato infection and acts downstream of the SA signaling pathway [104]. Therefore, the findings demonstrate a positive influence of silicon on modulating underlying molecular and biochemical processes in date palm in control or As stress conditions.

## 5. Conclusions

Conclusively, arsenic induced severe phytotoxicity and biochemical alterations in date palm, resulting in reduced biomass, photosynthetic pigments, and decreased levels of antioxidant enzymes. The enhanced levels of stress markers including superoxide anions and MDA contents further affirmed the stress induction of arsenic. However, silicon applications with or without arsenic treatment significantly recovered growth attributes. Overall, silicon protects against arsenic toxicity via limiting its translocation, hormonal modulation to regulate the antioxidant defense system. Moreover, anatomical observations revealed silicon’s positive influence on shoots and roots’ cellular morphology, with enhanced stomatal density in leaves. This study’s findings emphasize the significant role of silicon to support the growth attributes, reduce Arsenic toxicity in date palm, and further develop an understanding of arsenic threats in plants. 

## Data Availability

Not applicable.

## Supplementary Materials

The following supporting information can be downloaded at: https://www.mdpi.com/article/10.3390/plants11172263/s1, Figure S1: Influence of silicon and arsenic on size of stomata of date palm leaves, screened with Scan Electron Microscope. Silicon treated (Si+) Silicon non-treated (Si-), Silicon and Arsenic (Si+As) and Arsenic treated (As). Three biological replicates were screen for each sample; Figure S2: Influence of silicon and arsenic on leaf morphology and anatomy of date palm leaves, screened with Scan Electron Microscope. Silicon treated (Si+) Silicon non-treated (Si-), Silicon and Arsenic (Si+As) and Arsenic treated (As). Three biological replicates were screened for each sample. Table S1: Details of genes; name, description, product size, reference number, and oligonucleotide sequences used for qRT-PCR.

## References

[1] Etesami H. (2018). Bacterial mediated alleviation of heavy metal stress and decreased accumulation of metals in plant tissues: Mechanisms and future prospects. Ecotoxicol. Environ. Saf..

[2] Wu G., Kang H., Zhang X., Shao H., Chu L., Ruan C. (2010). A critical review on the bio-removal of hazardous heavy metals from contaminated soils: Issues, progress, eco-environmental concerns and opportunities. J. Hazard. Mater..

[3] Muchuweti M., Birkett J., Chinyanga E., Zvauya R., Scrimshaw M.D., Lester J. (2006). Heavy metal content of vegetables irrigated with mixtures of wastewater and sewage sludge in Zimbabwe: Implications for human health. Agric. Ecosyst. Environ..

[4] Kashif M., Sattar A., Ul-Allah S., Sher A., Ijaz M., Butt M., Qayyum A. (2021). Silicon Alleviates Arsenic Toxicity in Maize Seedlings by Regulating Physiological and Antioxidant Defense Mechanisms. J. Soil Sci. Plant Nutr..

[5] Salama K.F., Randhawa M.A., Al Mulla A.A., Labib O.A. (2019). Heavy metals in some date palm fruit cultivars in Saudi Arabia and their health risk assessment. Int. J. Food Prop..

[6] Shahid M., Dumat C., Khalid S., Schreck E., Xiong T., Niazi N.K. (2017). Foliar heavy metal uptake, toxicity and detoxification in plants: A comparison of foliar and root metal uptake. J. Hazard. Mater..

[7] Yadav S. (2010). Heavy metals toxicity in plants: An overview on the role of glutathione and phytochelatins in heavy metal stress tolerance of plants. S. Afr. J. Bot..

[8] Xu W., Dai W., Yan H., Li S., Shen H., Chen Y., Xu H., Sun Y., He Z., Ma M. (2015). Arabidopsis NIP3; 1 plays an important role in arsenic uptake and root-to-shoot translocation under arsenite stress conditions. Mol. Plant.

[9] Rahman M.M., Sengupta M.K., Ahamed S., Chowdhury U.K., Hossain M.A., Das B., Lodh D., Saha K.C., Pati S., Kaies I. (2005). The magnitude of arsenic contamination in groundwater and its health effects to the inhabitants of the Jalangi—one of the 85 arsenic affected blocks in West Bengal, India. Sci. Total Environ..

[10] Nickson R., McArthur J., Burgess W., Ahmed K.M., Ravenscroft P., Rahmann M. (1998). Arsenic poisoning of Bangladesh groundwater. Nature.

[11] Sun G. (2004). Arsenic contamination and arsenicosis in China. Toxicol. Appl. Pharmacol..

[12] Dai Y., Nasir M., Zhang Y., Gao J., Lv Y., Lv J. (2018). Comparison of DGT with traditional extraction methods for assessing arsenic bioavailability to Brassica chinensis in different soils. Chemosphere.

[13] Suriyagoda L.D., Dittert K., Lambers H. (2018). Arsenic in Rice soils and potential agronomic mitigation strategies to reduce arsenic bioavailability: A review. Pedosphere.

[14] Li Y., Ye F., Wang A., Wang D., Yang B., Zheng Q., Sun G., Gao X. (2016). Chronic arsenic poisoning probably caused by arsenic-based pesticides: Findings from an investigation study of a household. Int. J. Environ. Res. Public Health.

[15] Rahman Z., Singh V.P. (2019). The relative impact of toxic heavy metals (THMs)(arsenic (As), cadmium (Cd), chromium (Cr)(VI), mercury (Hg), and lead (Pb)) on the total environment: An overview. Environ. Monit. Assess..

[16] Mapa R.B. (2020). Environmental soil issues. The Soils of Sri Lanka.

[17] Abedi T., Mojiri A. (2020). Arsenic uptake and accumulation mechanisms in rice species. Plants.

[18] Rosas-Castor J., Guzmán-Mar J., Hernández-Ramírez A., Garza-González M., Hinojosa-Reyes L. (2014). Arsenic accumulation in maize crop (*Zea mays*): A review. Sci. Total Environ..

[19] O’Connor D., Peng T., Zhang J., Tsang D.C., Alessi D.S., Shen Z., Bolan N.S., Hou D. (2018). Biochar application for the remediation of heavy metal polluted land: A review of in situ field trials. Sci. Total Environ..

[20] Sharma S. (2022). Mechanisms of silicon for abiotic stress tolerance in higher plants: A review. Pharma Innov. J..

[21] Khaliq A., Ali S., Hameed A., Farooq M.A., Farid M., Shakoor M.B., Mahmood K., Ishaque W., Rizwan M. (2016). Silicon alleviates nickel toxicity in cotton seedlings through enhancing growth, photosynthesis, and suppressing Ni uptake and oxidative stress. Arch. Agron. Soil Sci..

[22] Ashfaque F., Inam A., Iqbal S., Sahay S. (2017). Response of silicon on metal accumulation, photosynthetic inhibition and oxidative stress in chromium-induced mustard (*Brassica juncea* L.). S. Afr. J. Bot..

[23] Khan A., Bilal S., Khan A.L., Imran M., Al-Harrasi A., Al-Rawahi A., Lee I.-J. (2020). Silicon-mediated alleviation of combined salinity and cadmium stress in date palm (*Phoenix dactylifera* L.) by regulating physio-hormonal alteration. Ecotoxicol. Environ. Saf..

[24] Shi Z., Yang S., Han D., Zhou Z., Li X., Liu Y., Zhang B. (2018). Silicon alleviates cadmium toxicity in wheat seedlings (*Triticum aestivum* L.) by reducing cadmium ion uptake and enhancing antioxidative capacity. Environ. Sci. Pollut. Res..

[25] Adrees M., Ali S., Rizwan M., Zia-ur-Rehman M., Ibrahim M., Abbas F., Farid M., Qayyum M.F., Irshad M.K. (2015). Mechanisms of silicon-mediated alleviation of heavy metal toxicity in plants: A review. Ecotoxicol. Environ. Saf..

[26] Bhat J.A., Shivaraj S., Singh P., Navadagi D.B., Tripathi D.K., Dash P.K., Solanke A.U., Sonah H., Deshmukh R. (2019). Role of silicon in mitigation of heavy metal stresses in crop plants. Plants.

[27] Gheshlaghpour J., Asghari B., Khademian R., Sedaghati B. (2021). Silicon alleviates cadmium stress in basil (*Ocimum basilicum* L.) through alteration of phytochemical and physiological characteristics. Ind. Crops Prod..

[28] Emamverdian A., Ding Y., Xie Y., Sangari S. (2018). Silicon mechanisms to ameliorate heavy metal stress in plants. BioMed Res. Int..

[29] Rizwan M., Meunier J.-D., Miche H., Keller C. (2012). Effect of silicon on reducing cadmium toxicity in durum wheat (*Triticum turgidum* L. cv. Claudio W.) grown in a soil with aged contamination. J. Hazard. Mater..

[30] Li L., Zheng C., Fu Y., Wu D., Yang X., Shen H. (2012). Silicate-mediated alleviation of Pb toxicity in banana grown in Pb-contaminated soil. Biol. Trace Elem. Res..

[31] de Jesus L.R., Batista B.L., da Silva Lobato A.K. (2017). Silicon reduces aluminum accumulation and mitigates toxic effects in cowpea plants. Acta Physiol. Plant..

[32] Tripathi D.K., Singh V.P., Kumar D., Chauhan D.K. (2012). Impact of exogenous silicon addition on chromium uptake, growth, mineral elements, oxidative stress, antioxidant capacity, and leaf and root structures in rice seedlings exposed to hexavalent chromium. Acta Physiol. Plant..

[33] Shehzad M., Rasheed H., Naqvi S.A., Al-Khayri J.M., Lorenzo J.M., Alaghbari M.A., Manzoor M.F., Aadil R.M. (2021). Therapeutic potential of date palm against human infertility: A review. Metabolites.

[34] Yaish M.W., Kumar P.P. (2015). Salt tolerance research in date palm tree (*Phoenix dactylifera* L.), past, present, and future perspectives. Front. Plant Sci..

[35] El-Juhany L.I. (2010). Degradation of date palm trees and date production in Arab countries: Causes and potential rehabilitation. Aust. J. Basic Appl. Sci..

[36] Abdulaal W.H., Zeyadi M., Baothman O.A., Zamzami M.A., Choudhry H., Almulaiky Y.Q., Saleh R.M., Mohamed S.A. (2017). Investigation of antioxidant and detoxifying capacities of some date cultivars (*Phoenix dactylifera* L.) irrigated with sewage water. RSC Adv..

[37] Al-Busaidi A., Shahroona B., Al-Yahyai R., Ahmed M. (2015). Heavy metal concentrations in soils and date palms irrigated by groundwater and treated wastewater. Pak. J. Agric. Sci..

[38] Chaâbene Z., Rorat A., Hakim I.R., Bernard F., Douglas G.C., Elleuch A., Vandenbulcke F., Mejdoub H. (2018). Insight into the expression variation of metal-responsive genes in the seedling of date palm (*Phoenix dactylifera*). Chemosphere.

[39] Hamid M.A. (2011). Growth and heavy metals uptake by date palm grown in mono-and dual culture in heavy metals contaminated soil. World Appl. Sci. J..

[40] Alansi S., Al-Qurainy F., Nadeem M., Khan S., Tarroum M., Alshameri A., Gaafar A.-R.Z. (2019). Cryopreservation: A tool to conserve date palm in Saudi Arabia. Saudi J. Biol. Sci..

[41] Mesnoua M., Roumani M., Mizab O., Zeguerrou R. (2020). Heavy metals differentially affect date palm pollen germination and tube elongation. Italus Hortus.

[42] Awad K.M., Salih A.M., Khalaf Y., Suhim A.A., Abass M.H. (2019). Phytotoxic and genotoxic effect of Aluminum to date palm (*Phoenix dactylifera* L.) in vitro cultures. J. Genet. Eng. Biotechnol..

[43] Bilal S., Khan A., Imran M., Khan A.L., Asaf S., Al-Rawahi A., Al-Azri M.S.A., Al-Harrasi A., Lee I.-J. (2022). Silicon-and Boron-Induced Physio-Biochemical Alteration and Organic Acid Regulation Mitigates Aluminum Phytotoxicity in Date Palm Seedlings. Antioxidants.

[44] Sumanta N., Haque C.I., Nishika J., Suprakash R. (2014). Spectrophotometric analysis of chlorophylls and carotenoids from commonly grown fern species by using various extracting solvents. Res. J. Chem. Sci..

[45] Cao Y.-Y., Yang M.-T., Chen S.-Y., Zhou Z.-Q., Li X., Wang X.-J., Bai J.-G. (2015). Exogenous sucrose influences antioxidant enzyme activities and reduces lipid peroxidation in water-stressed cucumber leaves. Biol. Plant..

[46] Bradford M.M. (1976). A rapid and sensitive method for the quantitation of microgram quantities of protein utilizing the principle of protein-dye binding. Anal. Biochem..

[47] Bilal S., Shahzad R., Lee I.-J. (2021). Synergistic interaction of fungal endophytes, Paecilomyces formosus LHL10 and Penicillium funiculosum LHL06, in alleviating multi-metal toxicity stress in *Glycine max* L.. Environ. Sci. Pollut. Res..

[48] Okaichi Y., Ishikura Y., Akimoto K., Kawashima H., Toyoda-Ono Y., Kiso Y., Okaichi H. (2005). Arachidonic acid improves aged rats’ spatial cognition. Physiol. Behav..

[49] Gajewska E., Skłodowska M. (2008). Differential biochemical responses of wheat shoots and roots to nickel stress: Antioxidative reactions and proline accumulation. Plant Growth Regul..

[50] Bilal S., Shahzad R., Khan A.L., Kang S.-M., Imran Q.M., Al-Harrasi A., Yun B.-W., Lee I.-J. (2018). Endophytic microbial consortia of phytohormones-producing fungus Paecilomyces formosus LHL10 and bacteria Sphingomonas sp. LK11 to Glycine max L. regulates physio-hormonal changes to attenuate aluminum and zinc stresses. Front. Plant Sci..

[51] Al-Harrasi A., Rehman N.U., Khan A.L., Al-Broumi M., Al-Amri I., Hussain J., Hussain H., Csuk R. (2018). Chemical, molecular and structural studies of Boswellia species: β-Boswellic Aldehyde and 3-epi-11β-Dihydroxy BA as precursors in biosynthesis of boswellic acids. PLoS ONE.

[52] Shahzad R., Khan A.L., Waqas M., Ullah I., Bilal S., Kim Y.-H., Asaf S., Kang S.-M., Lee I.-J. (2019). Metabolic and proteomic alteration in phytohormone-producing endophytic Bacillus amyloliquefaciens RWL-1 during methanol utilization. Metabolomics.

[53] Bilal S., Khan A.L., Shahzad R., Asaf S., Kang S.-M., Lee I.-J. (2017). Endophytic Paecilomyces formosus LHL10 augments Glycine max L. adaptation to Ni-contamination through affecting endogenous phytohormones and oxidative stress. Front. Plant Sci..

[54] Shahzad R., Waqas M., Khan A.L., Asaf S., Khan M.A., Kang S.-M., Yun B.-W., Lee I.-J. (2016). Seed-borne endophytic Bacillus amyloliquefaciens RWL-1 produces gibberellins and regulates endogenous phytohormones of Oryza sativa. Plant Physiol. Biochem..

[55] Liu L., Han R., Yu N., Zhang W., Xing L., Xie D., Peng D. (2018). A method for extracting high-quality total RNA from plant rich in polysaccharides and polyphenols using Dendrobium huoshanense. PLoS ONE.

[56] Silva R., Andreazza R., Da Ros C., Dellai A., Jacques R., Scheid D. (2015). Growth of tropical tree species and absorption of copper in soil artificially contaminated. Braz. J. Biol..

[57] Panaullah G.M., Alam T., Hossain M.B., Loeppert R.H., Lauren J.G., Meisner C.A., Ahmed Z.U., Duxbury J.M. (2009). Arsenic toxicity to rice (*Oryza sativa* L.) in Bangladesh. Plant Soil.

[58] Seyfferth A.L., Fendorf S. (2012). Silicate mineral impacts on the uptake and storage of arsenic and plant nutrients in rice (*Oryza sativa* L.). Environ. Sci. Technol..

[59] Muneer S., Park Y.G., Kim S., Jeong B.R. (2017). Foliar or subirrigation silicon supply mitigates high temperature stress in strawberry by maintaining photosynthetic and stress-responsive proteins. J. Plant Growth Regul..

[60] Nugues M., Roberts C. (2003). Coral mortality and interaction with algae in relation to sedimentation. Coral Reefs.

[61] Shri M., Kumar S., Chakrabarty D., Trivedi P.K., Mallick S., Misra P., Shukla D., Mishra S., Srivastava S., Tripathi R.D. (2009). Effect of arsenic on growth, oxidative stress, and antioxidant system in rice seedlings. Ecotoxicol. Environ. Saf..

[62] Duman F., Ozturk F., Aydin Z. (2010). Biological responses of duckweed (*Lemna minor* L.) exposed to the inorganic arsenic species As (III) and As (V): Effects of concentration and duration of exposure. Ecotoxicology.

[63] Stoeva N., Bineva T. (2003). Oxidative changes and photosynthesis in oat plants grown in As-contaminated soil. Bulg. J. Plant Physiol..

[64] Ma J.F., Yamaji N., Mitani N., Xu X.-Y., Su Y.-H., McGrath S.P., Zhao F.-J. (2008). Transporters of arsenite in rice and their role in arsenic accumulation in rice grain. Proc. Natl. Acad. Sci. USA.

[65] Tripathi P., Tripathi R.D., Singh R.P., Dwivedi S., Goutam D., Shri M., Trivedi P.K., Chakrabarty D. (2013). Silicon mediates arsenic tolerance in rice (*Oryza sativa* L.) through lowering of arsenic uptake and improved antioxidant defence system. Ecol. Eng..

[66] Ma J.F., Yamaji N. (2006). Silicon uptake and accumulation in higher plants. Trends Plant Sci..

[67] Anjum S.A., Tanveer M., Hussain S., Ashraf U., Khan I., Wang L. (2017). Alteration in growth, leaf gas exchange, and photosynthetic pigments of maize plants under combined cadmium and arsenic stress. Water Air Soil Pollut..

[68] Srivastava S., Sinha P., Sharma Y.K. (2017). Status of photosynthetic pigments, lipid peroxidation and anti-oxidative enzymes in Vigna mungo in presence of arsenic. J. Plant Nutr..

[69] Maglovski M., Gersi Z., Rybansky L., Bardacova M., Moravcikova J., Bujdos M., Dobrikova A., Apostolova E., Kraic J., Blehová A. (2019). Effects of nutrition on wheat photosynthetic pigment responses to arsenic stress. Pol. J. Environ. Stud..

[70] Kim Y.H., Khan A.L., Waqas M., Shim J.K., Kim D.H., Lee K.Y., Lee I.J. (2014). Silicon application to rice root zone influenced the phytohormonal and antioxidant responses under salinity stress. J. Plant Growth Regul..

[71] Tripathi R.D., Srivastava S., Mishra S., Singh N., Tuli R., Gupta D.K., Maathuis F.J. (2007). Arsenic hazards: Strategies for tolerance and remediation by plants. Trends Biotechnol..

[72] Mkandawire M., Lyubun Y.V., Kosterin P.V., Dudel E.G. (2004). Toxicity of arsenic species to *Lemna gibba* L. and the influence of phosphate on arsenic bioavailability. Environ. Toxicol. Int. J..

[73] Liang Y., Chen Q., Liu Q., Zhang W., Ding R. (2003). Exogenous silicon (Si) increases antioxidant enzyme activity and reduces lipid peroxidation in roots of salt-stressed barley (*Hordeum vulgare* L.). J. Plant Physiol..

[74] Kim Y.-H., Khan A.L., Waqas M., Lee I.-J. (2017). Silicon regulates antioxidant activities of crop plants under abiotic-induced oxidative stress: A review. Front. Plant Sci..

[75] Telles Nascimento K.J., Debona D., Silveira P.R., Silva L.C., DaMatta F.M., Rodrigues F.Á. (2016). Silicon-induced changes in the antioxidant system reduce soybean resistance to frogeye leaf spot. J. Phytopathol..

[76] Khan A., Bilal S., Khan A.L., Imran M., Shahzad R., Al-Harrasi A., Al-Rawahi A., Al-Azhri M., Mohanta T.K., Lee I.-J. (2020). Silicon and gibberellins: Synergistic function in harnessing ABA signaling and heat stress tolerance in date palm (*Phoenix dactylifera* L.). Plants.

[77] Khan T., Khan T., Hano C., Abbasi B.H. (2019). Effects of chitosan and salicylic acid on the production of pharmacologically attractive secondary metabolites in callus cultures of Fagonia indica. Ind. Crops Prod..

[78] Bari M., Prity S., Das U., Akther M., Sajib S., Reza M., Kabir A. (2020). Silicon induces phytochelatin and ROS scavengers facilitating cadmium detoxification in rice. Plant Biol..

[79] Dong Y., Xu L., Wang Q., Fan Z., Kong J., Bai X. (2014). Effects of exogenous nitric oxide on photosynthesis, antioxidative ability, and mineral element contents of perennial ryegrass under copper stress. J. Plant Interact..

[80] Seo M., Koshiba T. (2002). Complex regulation of ABA biosynthesis in plants. Trends Plant Sci..

[81] Qin X., Zeevaart J.A. (1999). The 9-cis-epoxycarotenoid cleavage reaction is the key regulatory step of abscisic acid biosynthesis in water-stressed bean. Proc. Natl. Acad. Sci. USA.

[82] Yan M., Yao Y., Mou K., Dan Y., Li W., Wang C., Liao W. (2022). The involvement of abscisic acid in hydrogen gas-enhanced drought resistance in tomato seedlings. Sci. Hortic..

[83] Abbasi N.A., Malik R.N., Frantz A., Jaspers V.L.B. (2016). A review on current knowledge and future prospects of organohalogen contaminants (OHCs) in Asian birds. Sci. Total Environ..

[84] Meguro A., Sato Y. (2014). Salicylic acid antagonizes abscisic acid inhibition of shoot growth and cell cycle progression in rice. Sci. Rep..

[85] Gao S., Yan R., Cao M., Yang W., Wang S., Chen F. (2008). Effects of copper on growth, antioxidant enzymes and phenylalanine ammonia-lyase activities in Jatropha curcas L. seedling. Plant Soil Environ..

[86] Vlot A.C., Dempsey D.M.A., Klessig D.F. (2009). Salicylic acid, a multifaceted hormone to combat disease. Annu. Rev. Phytopathol..

[87] Per T.S., Khan M.I.R., Anjum N.A., Masood A., Hussain S.J., Khan N.A. (2018). Jasmonates in plants under abiotic stresses: Crosstalk with other phytohormones matters. Environ. Exp. Bot..

[88] Kim Y.-H., Khan A.L., Kim D.-H., Lee S.-Y., Kim K.-M., Waqas M., Jung H.-Y., Shin J.-H., Kim J.-G., Lee I.-J. (2014). Silicon mitigates heavy metal stress by regulating P-type heavy metal ATPases, Oryza sativalow silicon genes, and endogenous phytohormones. BMC Plant Biol..

[89] Havshøi N.W., Fuglsang A.T. (2022). A critical review on natural compounds interacting with the plant plasma membrane H^+^-ATPase and their potential as biologicals in agriculture. J. Integr. Plant Biol..

[90] Chen W., Jia P.F., Yang W.C., Li H.J. (2020). Plasma membrane H^+^-ATPases-mediated cytosolic proton gradient regulates pollen tube growth. J. Integr. Plant Biol..

[91] Ding M., Zhang M., Zeng H., Hayashi Y., Zhu Y., Kinoshita T. (2021). Molecular basis of plasma membrane H^+^-ATPase function and potential application in the agricultural production. Plant Physiol. Biochem..

[92] Li J., Guo Y., Yang Y. (2022). The molecular mechanism of plasma membrane H^+^-ATPases in plant responses to abiotic stress. J. Genet. Genom..

[93] Nobori T. (2022). Closing the gap: A plasma membrane H^+^-ATPase regulates stomatal closure. Plant Cell.

[94] Lu Q., Chen S., Li Y., Zheng F., He B., Gu M. (2020). Exogenous abscisic acid (ABA) promotes cadmium (Cd) accumulation in Sedum alfredii Hance by regulating the expression of Cd stress response genes. Environ. Sci. Pollut. Res..

[95] Briat J.-F. (2010). Arsenic tolerance in plants:“Pas de deux” between phytochelatin synthesis and ABCC vacuolar transporters. Proc. Natl. Acad. Sci. USA.

[96] Larsen P.B., Geisler M.J., Jones C.A., Williams K.M., Cancel J.D. (2005). ALS3 encodes a phloem-localized ABC transporter-like protein that is required for aluminum tolerance in Arabidopsis. Plant J..

[97] Mishra S., Mattusch J., Wennrich R. (2017). Accumulation and transformation of inorganic and organic arsenic in rice and role of thiol-complexation to restrict their translocation to shoot. Sci. Rep..

[98] Yu S., Sun Q., Wu J., Zhao P., Sun Y., Guo Z. (2021). Genome-Wide Identification and Characterization of Short-Chain Dehydrogenase/Reductase (SDR) Gene Family in Medicago truncatula. Int. J. Mol. Sci..

[99] Lacombe E., Hawkins S., Van Doorsselaere J., Piquemal J., Goffner D., Poeydomenge O., Boudet A.M., Grima-Pettenati J. (1997). Cinnamoyl CoA reductase, the first committed enzyme of the lignin branch biosynthetic pathway: Cloning, expression and phylogenetic relationships. Plant J..

[100] Paddock T.N., Mason M.E., Lima D.F., Armstrong G.A. (2010). Arabidopsis protochlorophyllide oxidoreductase A (PORA) restores bulk chlorophyll synthesis and normal development to a porB porC double mutant. Plant Mol. Biol..

[101] Rahier A., Bergdoll M., Génot G., Bouvier F., Camara B. (2009). Homology Modeling and Site-Directed Mutagenesis Reveal Catalytic Key Amino Acids of 3 β-Hydroxysteroid-Dehydrogenase/C4-Decarboxylase from Arabidopsis. Plant Physiol..

[102] Sato Y., Morita R., Katsuma S., Nishimura M., Tanaka A., Kusaba M. (2009). Two short-chain dehydrogenase/reductases, NON-YELLOW COLORING 1 and NYC1-LIKE, are required for chlorophyll b and light-harvesting complex II degradation during senescence in rice. Plant J..

[103] González-Guzmán M., Apostolova N., Bellés J.M., Barrero J.M., Piqueras P., Ponce M.R., Micol J.L., Serrano R., Rodríguez P.L. (2002). The short-chain alcohol dehydrogenase ABA2 catalyzes the conversion of xanthoxin to abscisic aldehyde. Plant Cell.

[104] Hwang S.-G., Lin N.-C., Hsiao Y.-Y., Kuo C.-H., Chang P.-F., Deng W.-L., Chiang M.-H., Shen H.-L., Chen C.-Y., Cheng W.-H. (2012). The Arabidopsis short-chain dehydrogenase/reductase 3, an abscisic acid deficient 2 homolog, is involved in plant defense responses but not in ABA biosynthesis. Plant Physiol. Biochem..

