# Supplementary material for: Silicon-Induced Tolerance against Arsenic Toxicity by Activating Physiological, Anatomical and Biochemical Regulation in Phoenix dactylifera (Date Palm)
Source: Plants (Basel). 2022 Aug 31;11(17):2263. doi: 10.3390/plants11172263 (PMC9459973; doi:10.3390/plants11172263)

Silicon Induced Tolerance Against Arsenic Toxicity By Activating Physiological, Anatomical And Biochemical Regulation In *Phoenix dactylifera* (Date Palm)

Taimoor Khan[1], Saqib Bilal[1*], Sajjad Asaf[1], Safiya Salim Alamri[1], Muhammad Imran[2], Abdul Latif Khan[3*], Ahmed Al-Rawahi[1], In-Jung Lee[3] and Ahmed Al-Harrasi[1*]

Table S1. Details of genes; name, description, product size, reference number, and oligonucleotide sequences used for qRT-PCR.

Gene name	Description	Primer sequence (5'-3')
LSi-1	Low silicon 1 related gene	F: AGTCGATCTCGGTCTCGTCA<br>R: AGCCCCGGAATTAACAGAGC
SOD	Superoxide dismutase [Fe], chloroplastic	F: TGGCTGGCAAAGAGCATGTA R: R: CTCGAGCCGTTGCTATGTGA
NCED1	9-cis-epoxycarotenoid dioxygenase,	F: GCCTCCTCGCACCACTATTC<br>R: CGGGTCGATGAAGTTGTTGA
ZEP	zeaxanthin epoxidase	F: CCACTCTTTCCTCGCCAATC<br>R: GAATCTGGCAATGGGCTTTC
SDR	Phoenix dactylifera short chain dehydrogenase/reductase	F: GCTGGTGTTGAAGGGAGGAG<br>R: GGTTTTGTCCGGTGACGAAC
ALMT-1	Phoenix dactylifera aluminum-activated malate transporter 1-like	F: CCGGCGAATTAAGAAGCACG<br>R: TAACGGTGGCCTTGGGAATC
ALMT2	Phoenix dactylifera aluminum-activated malate transporter 1-like	F: GCTCTTTGGTGGCTCTTTTG<br>R: CGAGAATATGTGGCCATTCC
PPMA-3	Phoenix dactylifera plasma membrane ATPase-3	F: TGGATTTCTGCTCACTCCCAC<br>R: CTTCTCTACTGCGGCTGAGGA
PPMA-4	Phoenix dactylifera plasma membrane ATPase-4	F: TTGAGGTGACGACCAAGAGC<br>R: GAAACCGGCTACAGGACCAA
Act	Phoenix dactylifera actin	F: ATTCTGCAGGACTGGGAAGC<br>R: CGAACAGGTTGGCTGAATTGT

Supplementary figure S1: Influence of silicon and arsenic on size of stomata of date palm leaves, screened with Scan Electron Microscope. Silicon treated (Si+) Silicon non-treated (Si-), Silicon and Arsenic (Si+As) and Arsenic treated (As). Three biological replicates were screen for each sample

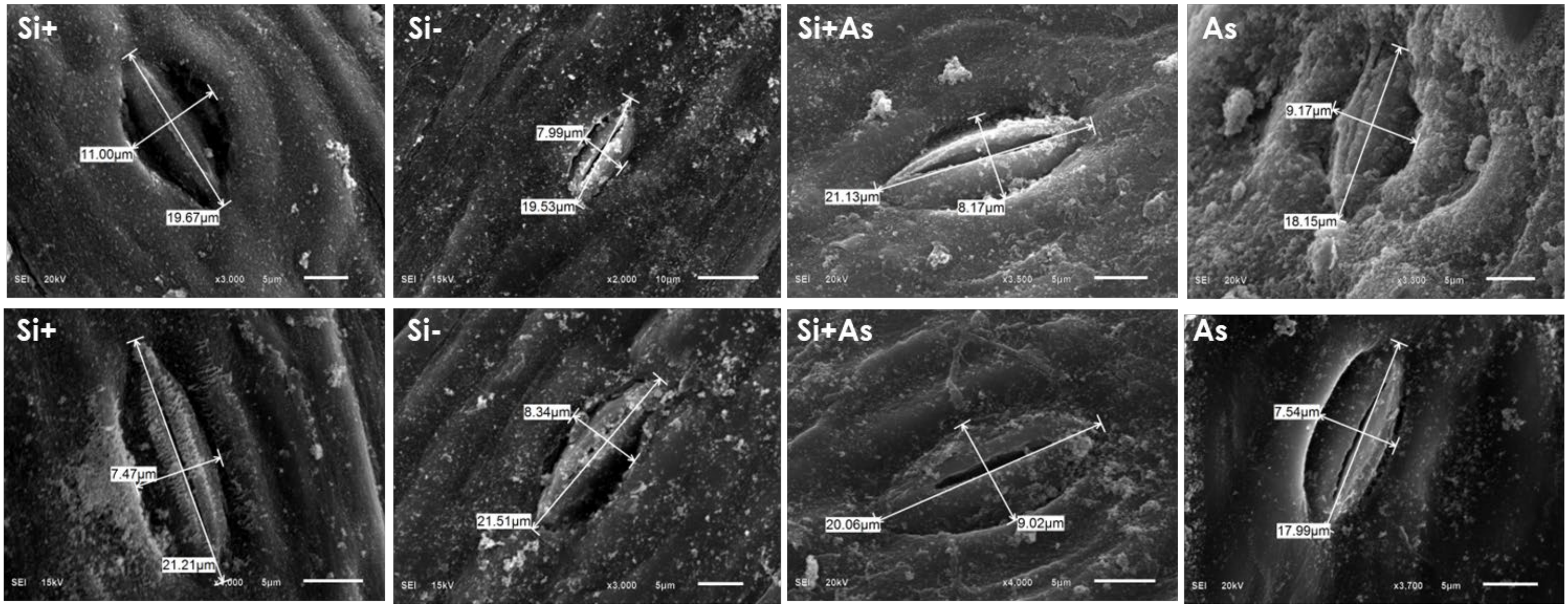

Supplementary figure S2: Influence of silicon and arsenic on leaf morphology and anatomy of date palm leaves, screened with Scan Electron Microscope. Silicon treated (Si+) Silicon non-treated (Si-), Silicon and Arsenic (Si+As) and Arsenic treated (As). Three biological replicates were screened for each sample.

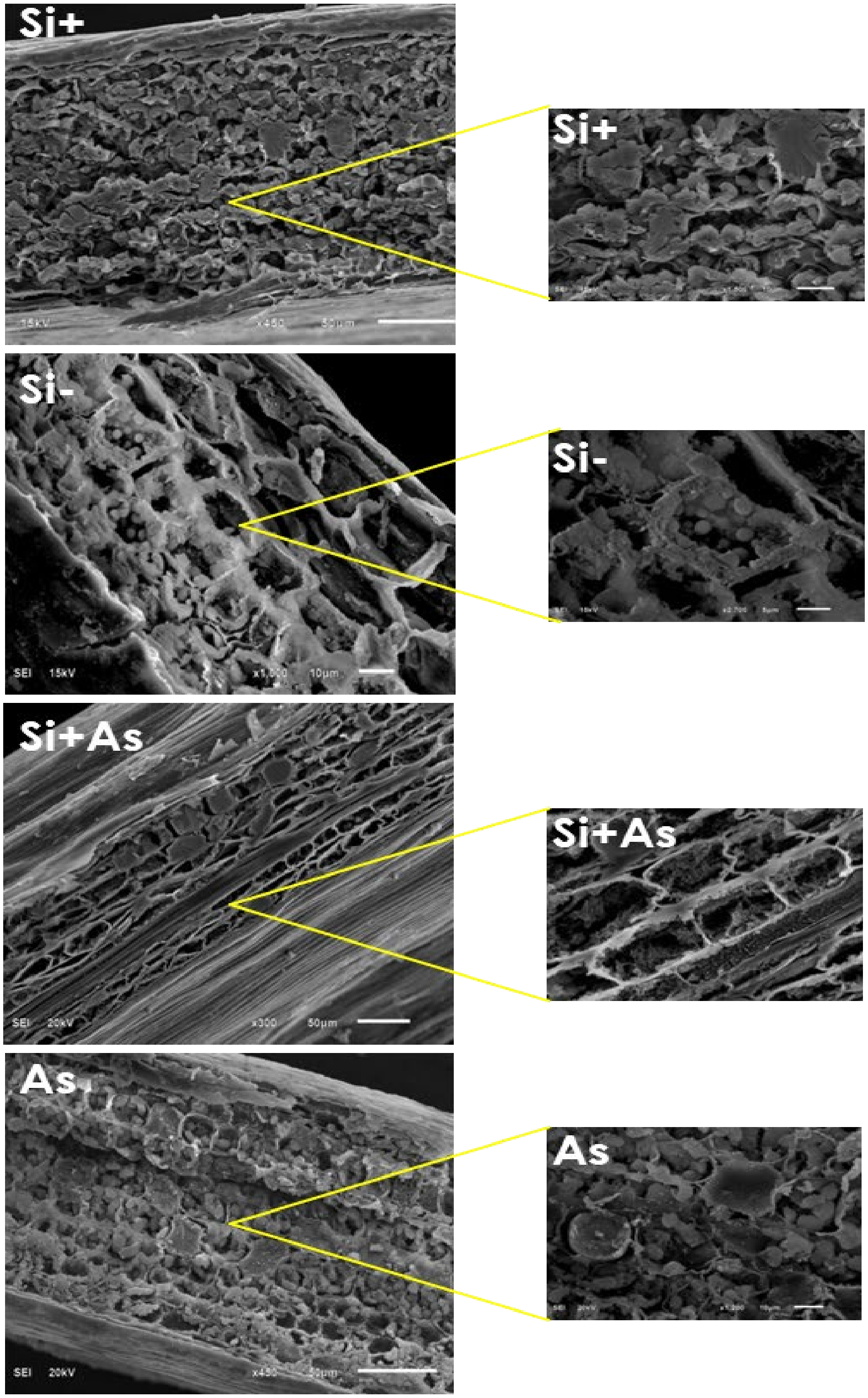

Supplement: Supplementary file 1 [file plants-11-02263-s001.zip › plants-1859645-supplementary material.pdf]
